# Secondary structure transitions and dual PIP2 binding define cardiac KCNQ1-KCNE1 channel gating

**DOI:** 10.1038/s41422-025-01182-9

**Published:** 2025-10-02

**Authors:** Ling Zhong, Xiaoqing Lin, Xinyu Cheng, Shuangyan Wan, Yaoguang Hua, Weiwei Nan, Bin Hu, Xiangjun Peng, Zihan Zhou, Qiansen Zhang, Huaiyu Yang, Frank Noé, Zhenzhen Yan, Dexiang Jiang, Hangyu Zhang, Fengjiao Liu, Chenxin Xiao, Zhuo Zhou, Yimin Mou, Haijie Yu, Lijuan Ma, Chen Huang, Vincent Kam Wai Wong, Sookja Kim Chung, Bing Shen, Zhi-Hong Jiang, Erwin Neher, Wandi Zhu, Jin Zhang, Panpan Hou

**Affiliations:** 1 Dr. Neher’s Biophysics Laboratory for Innovative Drug Discovery; State Key Laboratory of Mechanism and Quality of Chinese Medicine & School of Pharmacy, Faculty of Medicine; Faculty of Chinese Medicine, Macau University of Science and Technology, Macau SAR, China; 2 The MOE Basic Research and Innovation Center for the Targeted Therapeutics of Solid Tumors, The Second Affiliated Hospital, Jiangxi Medical College, Nanchang University, Nanchang, Jiangxi China; 3 Jiangxi Provincial Key Laboratory of Tumor Biology, School of Basic Medical Sciences, Jiangxi Medical College, Nanchang University, Nanchang, Jiangxi China; 4 School of Basic Medical Sciences, Gannan Medical University, Ganzhou, Jiangxi China; 5 Department of Engineering Mechanics, Applied Mechanics Laboratory, Institute of Biomechanics and Medical Engineering, Tsinghua University, Beijing, China; 6 Shanghai Key Laboratory of Regulatory Biology, Institute of Biomedical Sciences and School of Life Sciences, East China Normal University, Shanghai, China; 7 Department of Mathematics and Informatics, Freie Universität Berlin, Berlin, Germany; 8 Macau University of Science and Technology Innovation Technology Research Institute, Hengqin, Guangdong, China; 9 State Key Laboratory of Pharmaceutical Biotechnology, The University of Hong Kong, Hong Kong, China; 10 College of Medicine, Department of Molecular Medicine and Therapeutics, The Ohio State University. Columbus, OH, USA

**Keywords:** Cryoelectron microscopy, Single-molecule biophysics

## Abstract

The KCNQ1 + KCNE1 potassium channel complex produces the slow delayed rectifier current (I_Ks_) critical for cardiac repolarization. Loss-of-function mutations in *KCNQ1* and *KCNE1* cause long QT syndrome (LQTS) types 1 and 5 (LQT1/LQT5), accounting for over one-third of clinical LQTS cases. Despite prior structural work on KCNQ1 and KCNQ1 + KCNE3, the structural basis of KCNQ1 + KCNE1 remains unresolved. Using cryo-electron microscopy and electrophysiology, we determined high-resolution (2.5–3.4 Å) structures of human KCNQ1_APO_, and KCNQ1 + KCNE1 in both closed and open states. KCNE1 occupies a pivotal position at the interface of three KCNQ1 subunits, inducing six helix-to-loop transitions in KCNQ1 transmembrane segments. Three of them occur at both ends of the S4–S5 linker, maintaining a loop conformation during I_Ks_ gating, while the other three, in S6 and helix A, undergo dynamic helix-loop transitions during I_Ks_ gating. These structural rearrangements: (1) stabilize the closed pore and the conformation of the intermediate state voltage-sensing domain, thereby determining channel gating, ion permeation, and single-channel conductance; (2) enable a dual-PIP2 modulation mechanism, where one PIP2 occupies the canonical site, while the second PIP2 bridges the S4–S5 linker, KCNE1, and the adjacent S6’, stabilizing channel opening; (3) create a fenestration capable of binding compounds specific for KCNQ1 + KCNE1 (e.g., AC-1). Together, these findings reveal a previously unrecognized large-scale secondary structural transition during ion channel gating that fine-tunes I_Ks_ function and provides a foundation for developing targeted LQTS therapy.

## Introduction

The KCNQ1 + KCNE1 channel complex serves as a master regulator of cardiac repolarization and electrical stability of the heart.^1–4^ Dysfunctional variants in either the pore-forming subunit KCNQ1 or the auxiliary subunit KCNE1 are directly implicated in long QT syndrome (LQTS) types 1 and 5 (LQT1 and LQT5), two leading causes of sudden cardiac death in young individuals.^5–7^ The cardiac-specific KCNE1 auxiliary subunit (also known as MinK) is a single transmembrane protein consisting of 129 amino acids.^1–4,8^ When co-assembled with KCNQ1 to form the KCNQ1 + KCNE1 (I_Ks_) channel,^3,4,9^ this heteromeric complex dynamically orchestrates ventricular action potential termination through three key features: (1) slowed activation gating kinetics. KCNE1 significantly increases macroscopic current amplitude and single-channel conductance, slows activation and deactivation kinetics, and right-shifts the voltage-dependent activation^10–17^; (2) altered ion selectivity. KCNE1 reduces the rubidium/potassium (Rb^+^/K^+^) permeability ratio through the selectivity filter (SF)^16,18–21^; and (3) changed pharmacological profile. KCNE1 modulates the channel’s sensitivity to activators (e.g., ML277, polyunsaturated fatty acids) and blockers (e.g., XE991, AC-1).^16,18,22–29^ Despite decades of functional electrophysiology characterization, the structural basis for profound modulation of KCNQ1 by KCNE1 remains elusive. Addressing this gap is critical for revealing the mechanisms underlying I_Ks_ channel allosteric gating and enabling structure-guided therapeutics development for LQTS.

The KCNQ1 channel (K_V_7.1/K_V_LQT1) is a voltage-gated potassium channel,^3,4,9^ with a canonical tetrameric architecture.^30–34^ Each subunit comprises six transmembrane segments (S1–S6), organized into four peripheral voltage-sensing domains (VSDs, S1–S4) and a central pore domain (PD, S5–S6). The VSDs are connected to the PD via S4–S5 linkers. KCNQ1 exhibits a domain-swapped architecture where each VSD is in close contact with a neighboring subunit’s PD.^30–34^ Activation of KCNQ1 follows a hand-and-elbow gating mechanism involving sequential VSD-to-pore transitions^23^: (1) S4 transition from resting to intermediate state triggers intra-subunit “hand” interactions (between S4–S5 linker and lower S6), producing an intermediate open (IO) state; (2) subsequent S4 transition to activated state engages inter-subunit “elbow” interactions (between the S4/S4–S5 linker and the neighboring pore), yielding the activated open (AO) state.^11,16,18,23,30,35–37^ Notably, the KCNE1 association selectively suppresses the IO state but enhances the AO state, restricting the KCNQ1 + KCNE1 channel only to the AO state.^16,18,23,30,36,37^ Although this allosteric regulation is functionally defined, it lacks structural evidence, which leaves several key questions unresolved. How does a small KCNE1 subunit completely reshape KCNQ1’s conformational changes during gating? What structural determinants underlie I_Ks_’s function distinct from KCNQ1 alone and the epithelial KCNQ1 + KCNE3 channel that is constitutively open?^8,10,13,35,38,39^

In this study, we integrated cryo-electron microscopy (cryo-EM) and electrophysiology to elucidate the structural basis of regulation of KCNQ1 by KCNE1 that shapes cardiac repolarization. We determined high-resolution (2.5–3.4 Å) structures of the human KCNQ1 in apo state (closed), and KCNQ1 + KCNE1 complex in apo state (closed) and PIP2-bound open state. Detailed analyses reveal that KCNE1 occupies a strategic position at the interface of three KCNQ1 subunits, inducing six secondary structural helix-to-loop transitions in KCNQ1 transmembrane segments, which: (1) stabilize the closed pore and intermediate state VSD, thereby determining channel gating, ion permeation, and single-channel conductance; (2) establish a dual-PIP2 modulation mechanism, where one PIP2 occupies the canonical site, while the second PIP2 bridges the S4–S5 linker, KCNE1, and adjacent S6’, stabilizing channel opening; (3) create a fenestration that allows binding of compounds to selectively modulate KCNQ1 + KCNE1 activity. Taken together, our findings provide a new structural paradigm for ion channel gating, and establish a structural framework for developing targeted therapies against LQTS.

## Results

### Structure of the KCNQ1 + KCNE1 channel

The KCNQ1 activation currents can be fitted by a double exponential function, with the fast (τ_-f_) and slow (τ_-s_) components representing the currents of the IO and AO states, respectively^11,18,23,30,35–37^ (Supplementary information, Fig. S1a). The association of KCNE1 profoundly modulates KCNQ1 channel gating properties, which: (1) increases the current amplitude, along with slowed activation kinetics (Supplementary information, Fig. S1a); (2) right-shifts the voltage-dependent activation by ~70 mV (Supplementary information, Fig. S1c); (3) suppresses the IO current, so that the KCNQ1 + KCNE1 channel opens only in the slow-activating AO state (Supplementary information, Fig. S1a).

To elucidate the structural basis of these intensive modulations by KCNE1, we determined cryo-EM structures of human KCNQ1 + KCNE1 channel under different conditions. Initial attempts with full-length human KCNQ1 co-expressed with KCNE1 yielded only KCNQ1 protein lacking KCNE1 after purification (Supplementary information, Fig. S2), likely due to the structural flexibility of the N- and C-termini of KCNQ1.^31,32^ We therefore engineered a stabilized construct (KCNQ1 + KCNE1)_EM_, fusing KCNE1 to N/C-termini-truncated KCNQ1 (KCNE1-∆N/∆C/KCNQ1). A similar strategy has been widely used in both structural and functional studies.^31,32,40^ This (KCNQ1 + KCNE1)_EM_ construct produced channels exhibiting almost identical current kinetics and G–V relation to the wild-type (WT) KCNQ1 + KCNE1 channel (Supplementary information, Fig. S1b, c). SDS-PAGE results showed that the (KCNQ1 + KCNE1)_EM_ protein was successfully purified with size exclusion chromatography (Supplementary information, Fig. S1d, e).

**Fig. 1:**
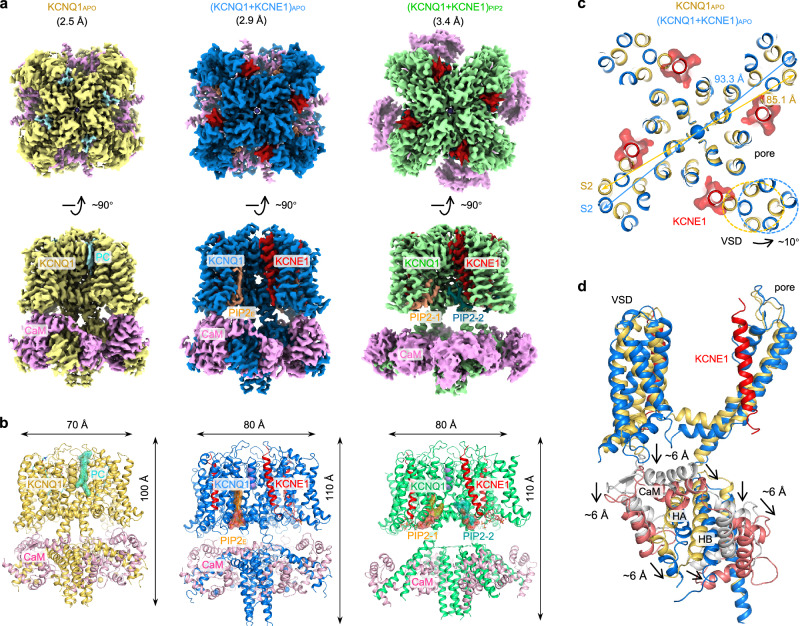
Overall closed- and open-state structures of KCNQ1 + KCNE1. **a** Top and side views of KCNQ1 density maps in the apo condition, and (KCNQ1 + KCNE1)_EM_ density maps in apo and in the presence of PIP2. An endogenous PC molecule was observed in KCNQ1_APO_, and an endogenous PIP2 molecule was observed in (KCNQ1 + KCNE1)_APO_, while two PIP2 molecules, PIP2-1 and PIP2-2, were observed in each subunit of (KCNQ1 + KCNE1)_PIP2_. Color codes: KCNQ1_APO_ (yellow), (KCNQ1 + KCNE1)_APO_ (blue), (KCNQ1 + KCNE1)_PIP2_ (green), KCNE1 (red), PIP2-1 (orange), PIP2-2 (dark green), and CaM (pink). **b** Structure models of KCNQ1_APO_, (KCNQ1 + KCNE1)_APO_, and (KCNQ1 + KCNE1)_PIP2_. **c** KCNE1-induced horizontal expansion of the VSD (from 85.1 Å to 93.3 Å for S2–I161), and ~10° rotation of the VSD (counterclockwise, based on the residue I132 from the middle of S1 to the center pore axis). Structures were aligned to the filter. **d** KCNE1-induced vertical expansions of the S2–S3 linker and the cytosolic domain (~6 Å). For clarity, CaM in the KCNQ1_APO_ was colored gray, and CaM in the (KCNQ1 + KCNE1)_APO_ was colored rust red.

We determined high-resolution structures of human KCNQ1 in the apo state: KCNQ1_APO_ (2.5 Å), and KCNQ1 + KCNE1 complex in both the apo state: (KCNQ1 + KCNE1)_APO_ (2.9 Å) and in the presence of PIP2: (KCNQ1 + KCNE1)_PIP2_ (3.4 Å) (Fig. 1a and Supplementary information, Figs. S3–S5). All structures were determined with C4 symmetry. The KCNQ1_APO_ structure, serving as a reference structure, aligned well with previous studies,^32^ but revealed a previously unrecognized lipid molecule bound in the cleft formed by three different subunits at the extracellular side (cyan, Fig. 1a, b and Supplementary information, Fig. S3). The EM map resolved this lipid density, which matches phosphatidylcholine (PC) (Fig. 1a, b and Supplementary information, Fig. S3), the predominant phospholipid in the outer leaflet of the plasma membrane.^41^ Interestingly, in both (KCNQ1 + KCNE1)_APO_ and (KCNQ1 + KCNE1)_PIP2_ structures, the KCNE1 transmembrane segment (D39–I66 of KCNE1 for (KCNQ1 + KCNE1)_APO_, and D39–S68 of KCNE1 for (KCNQ1 + KCNE1)_PIP2_) occupies the same cleft, in place of the PC molecule (Fig. 1a, b). The remaining regions of KCNE1 were not resolved, presumably also due to their structural flexibility.

The KCNQ1 + KCNE1 structure adopts a domain-swapped architecture (Supplementary information, Fig. S6). Each tetrameric channel can bind four KCNE1 subunits, following a KCNQ1:KCNE1 stoichiometry of 4:4 (Fig. 1a, b). The 4:4 stoichiometry was confirmed by symmetry-free refinement (Supplementary information, Fig. S7). Structural analysis of KCNQ1_APO_ and (KCNQ1 + KCNE1)_APO_, aligned to the SF, shows remarkable KCNE1-induced structural changes of KCNQ1: (1) the KCNE1 association induces a significant ~10° rotation (counterclockwise from top view) of the VSD relative to the pore (Fig. 1c). As a result, the VSD undergoes a clear horizontal expansion (e.g., S2–I161: 85.1 → 93.3 Å, Fig. 1c), and the S1 segment shows 3.6–8.5 Å displacements (K121, I132, and S143 from bottom, middle, and top of S1 showed 8.5 Å, 7.7 Å, and 3.6 Å displacements, respectively) (Supplementary information, Fig. S8). (2) KCNE1 also induces a downward movement of the S2–S3 linker, the S4–S5 linker, and the entire cytosolic domain by ~6 Å (Fig. 1d). Consequently, KCNE1 induced a global structural expansion of almost every amino acid of KCNQ1 (Supplementary information, Videos S1, S2).

### The KCNQ1 and KCNE1 interfaces

**Fig. 2:**
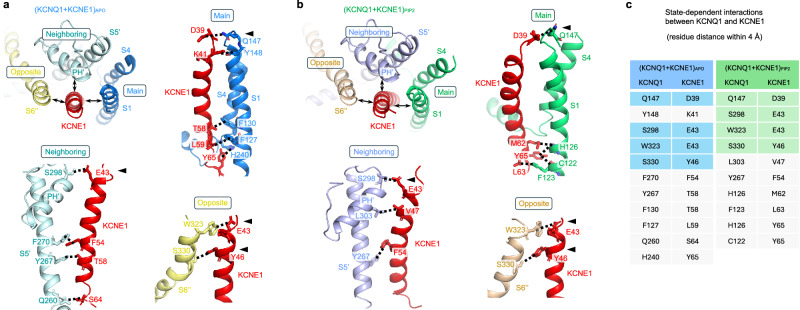
Interaction interfaces between KCNQ1 and KCNE1. **a** In (KCNQ1 + KCNE1)_APO_ structure, KCNE1 shows direct contact with three different subunits: the main subunit (main), its neighboring subunit (neighboring), and its opposite subunit (opposite). Between “main” and KCNE1: Q147/D39, Y148/K41, F130/T58, F127/L59 and H240/Y65; between “neighboring” and KCNE1: S298/E43, F270/F54, Y267/T58 and Q260/S64; between “opposite” and KCNE1: W323/E43 and S330/Y46. **b** In (KCNQ1 + KCNE1)_PIP2_ structure, KCNE1 shows direct contact with three different subunits: the main subunit (main), its neighboring subunit (neighboring), and its opposite subunit (opposite). Between “main” and KCNE1: Q147/D39, H126/M62, H126/Y65, C122/Y65, and F123/L63; between “neighboring” and KCNE1: S298/E43, L303/V47 and Y267/F54; between “opposite” and KCNE1: W323/E43 and S330/Y46. **c** State-dependent interactions between KCNQ1 and KCNE1 in (KCNQ1 + KCNE1)_APO_ and (KCNQ1 + KCNE1)_PIP2_. Among them, four residue pairs Q147/D39, S298/E43, W323/E43 and S330/Y46, remain interacted in both (KCNQ1 + KCNE1)_APO_ and (KCNQ1 + KCNE1)_PIP2_ (marked with black triangles in **a**, **b**), and are highlighted with blue and green colors, respectively.

The (KCNQ1 + KCNE1)_APO_ and (KCNQ1 + KCNE1)_PIP2_ structures adopt closed and open conformations, respectively, as detailed in later sections. These structures provide new mechanistic insights into how KCNE1 modulates KCNQ1 gating. KCNE1 is critically positioned to directly contact: (1) the VSD of one subunit (the “main” subunit), (2) the PD (S5’ and pore helix’, PH’) of its adjacent subunit (the “neighboring” subunit), and (3) the PD (S6”, yellow) of the diagonally opposed subunit (the “opposite” subunit; Fig. 2a, b). This tripartite binding geometry allows KCNE1 to simultaneously engage multiple functional modules of KCNQ1.

In the closed-state (KCNQ1 + KCNE1)_APO_ structure, KCNE1 forms extensive interactions with all three subunits: (1) Main subunit: strong contacts with both top and bottom parts of S1 (KCNQ1/KCNE1 residue pairs Q147/D39, Y148/K41, F130/T58, and F127/L59), and with the bottom part of S4 (H240/Y65). (2) Neighboring subunit: interactions with the PH’ (S298/E43), middle S5’ (F270/F54 and Y267/T58), and bottom S5’ (Q260/S64). (3) Opposite subunit: engagement of S6” (W323/E43 and S330/Y46) (Fig. 2a, c).

Upon PIP2-induced channel opening, in (KCNQ1 + KCNE1)_PIP2_, these interactions exhibit significant remodeling: (1) Main subunit: the Q147/D39 interaction is retained, while new contacts form (H126/M62, H126/Y65, C122/Y65, and F123/L63) between S1 and KCNE1. (2) Neighboring subunit: the S298/E43 interaction persists, with additional bonds forming at PH’ (L303/V47) and S5’ (Y267/F54). (3) Opposite subunit: key interactions (W323/E43, S330/Y46) remain stable (Fig. 2b, c).

These state-dependent interactions in (KCNQ1 + KCNE1)_APO_ and (KCNQ1 + KCNE1)_PIP2_ highlight how KCNE1 dynamically modulates KCNQ1’s global conformation and gating.

### KCNE1 stabilizes the intermediate state VSD of KCNQ1

The voltage-dependent activation of KCNQ1 + KCNE1 channels proceeds through three sequential steps: VSD activation, VSD–pore coupling, and pore opening. We next aim to elucidate the structural basis of KCNQ1 + KCNE1 gating process.

To document the effect of KCNE1-induced conformational changes on VSD activation, we aligned (KCNQ1 + KCNE1)_APO_ and (KCNQ1 + KCNE1)_PIP2_ structures to the SF of KCNQ1_APO_. The remarkable counterclockwise rotation and horizontal expansion of the VSD strongly suggest that KCNE1 may alter the VSD activation.

**Fig. 3:**
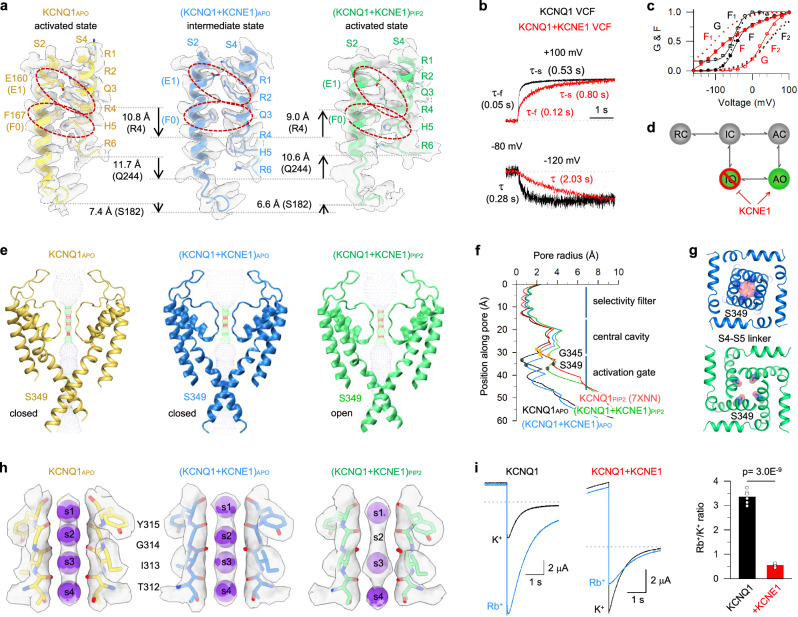
KCNE1-induced structural changes of the KCNQ1 VSD and pore. **a** Structural comparison of VSDs among KCNQ1_APO_, (KCNQ1 + KCNE1)_APO_, and (KCNQ1 + KCNE1)_PIP2_. Only S2 and S4 were shown for clarity. Red circles highlight E160/R231 and F167/Q234 interactions in (KCNQ1 + KCNE1)_APO_, and E160/R237 and F167/H240 interactions in KCNQ1_APO_ and (KCNQ1 + KCNE1)_PIP2_. Arrows indicate movements of R4, the S4 loop, and the S2 loop. Density maps are shown in transparent gray. **b** VCF results of KCNQ1 (black) and KCNQ1 + KCNE1 (red). VCF traces recorded at +100 mV and –120 mV were normalized to compare their activation and deactivation. **c** Normalized fluorescence intensity (F) and conductance (G). The F–V relationships (filled black circles for KCNQ1, and filled red circles for KCNQ1 + KCNE1) were fitted with a double Boltzmann equation (F_1_ and F_2_ components, black dotted lines for KCNQ1, and red dotted lines for KCNQ1 + KCNE1), and the G–V relationships were fitted with a single Boltzmann equation (open black circles for KCNQ1, and filled red circles for KCNQ1 + KCNE1). All *n* ≥ 5. **d** Cartoon scheme showing that KCNE1 suppresses IO state but enhances AO state. **e**, **f** Pore radius analysis of KCNQ1_APO_, (KCNQ1 + KCNE1)_APO_, and (KCNQ1 + KCNE1)_PIP2_ with front and back subunits excluded for clarity. Black and orange dots indicate the residues S349 and G345, respectively. **g** Structural comparison of the activation gate (S349) between (KCNQ1 + KCNE1)_APO_ and (KCNQ1 + KCNE1)_PIP2_. **h** Structural comparison of SF among KCNQ1_APO_, (KCNQ1 + KCNE1)_APO_, and (KCNQ1 + KCNE1)_PIP2_. The s2 K^+^ binding site was disrupted in (KCNQ1 + KCNE1)_PIP2_. Density maps are shown in transparent gray. **i** Representative currents of KCNQ1 and KCNQ1 + KCNE1 in the presence of 100 mM Rb^+^ or 100 mM K^+^ extracellular solutions. The dashed lines indicate zero current. Currents were tested at 40 mV for 4 s and then stepped to –60 mV to record tail currents. The Rb^+^/K^+^ ratio was 3.3 ± 0.1 for KCNQ1 (*n* = 6) and 0.6 ± 0.1 for KCNQ1 + KCNE1 (*n* = 6, *P* = 3.0E-9 with *t*-test).

Activation of KCNQ1 VSD involves coordinated interactions between gating charge residues (R1–R6) on S4 and conserved residues, including E160 (E1) from S2, and the charge transfer center (F167 (F0) together with E170 and D202^42,43^). In the intermediate state VSD, E1 pairs with the second gating charge residue R231 (R2), and F0 interacts with Q234 (Q3); while in the activated state VSD, E1 engages with the fourth gating charge residue R237 (R4) and F0 interacts with H240 (H5).^11,12,16,35^ Similar results were observed in our KCNQ1_APO_ structure (Fig. 3a), confirming its activated state of VSD. This established KCNQ1 VSD activation pattern offers a reliable reference for validating VSD states captured in (KCNQ1 + KCNE1)_APO_ and (KCNQ1 + KCNE1)_PIP2_ structures. These two structures exhibit distinct VSD conformations: in (KCNQ1 + KCNE1)_APO_, E1 and F0 are in close contact with R2 and Q3, respectively (Fig. 3a), which strongly suggests that the VSD is in the intermediate state; while in (KCNQ1 + KCNE1)_PIP2_, E1 and F0 are clearly pointing to R4 and H5, respectively (Fig. 3a), indicating that its VSD is in the activated state.

Voltage-clamp fluorometry (VCF) provides a powerful tool to track VSD activation by fluorescence.^11,12,16,44^ From time dependence of VCF results, the VSD activations (from holding –80 mV to +100 mV) of KCNQ1 and KCNQ1 + KCNE1 both display biphasic kinetics^11^ (τ_-f_ and τ_-s_), with KCNQ1 + KCNE1 exhibiting a much slower time course (Fig. 3b). Similar results were observed for VSD deactivations (from holding –80 mV to –120 mV), with KCNQ1 + KCNE1 also showing significantly slower time course (Fig. 3b). Both results suggest that KCNE1 stabilizes the intermediate state VSD. From voltage dependence, the fluorescence–voltage (F–V) relationships could be well-fitted by a double Boltzmann function comprising two components, F_1_ and F_2_. F_1_ corresponds to the VSD transition from the resting state to the intermediate state, and F_2_ reflects the VSD transition from the intermediate state to the activated state^11,18,23,30^ (Fig. 3c). However, KCNE1 stabilizes the intermediate state VSD (i.e., intermediate-closed or IC state) and suppresses its coupling to the pore^16,18,23,30,36,37^ (Fig. 3c). As a result, the channel opening is coupled to F_2_, allowing it to open exclusively in the AO state (Fig. 3c, d).

These VCF results suggest that although KCNQ1 + KCNE1 still shares the same VSD activation pattern as KCNQ1, detailed structures in the intermediate and activated states might be different. In line with this, we aligned the VSD structures to S1–S2 and observed conformational changes of S3–S4: compared to the intermediate state VSD structures of KCNQ1 (PDB: 6MIE^35^ and 8SIM^34^), the S3–S4 of (KCNQ1 + KCNE1)_APO_ exhibited ~3.5 Å displacements (Supplementary information, Fig. S9), whereas the VSD of (KCNQ1 + KCNE1)_PIP2_ preserved overall S1–S4 positions, compared to the activated state VSD of KCNQ1 (PDB: 7XNL^30^ and 8SIK^34^) (Supplementary information, Fig. S9). These KCNE1-induced VSD rearrangements together with the ~10° rotation, likely contribute to the stabilization of the intermediate state and the markedly slower VSD activation kinetics.

### KCNE1 induces structural remodeling of the ion conduction pathway

We next investigated the KCNE1-induced structural remodeling of the PD of KCNQ1. Using HOLE program analysis,^45^ we performed pore radius analysis across KCNQ1_APO_, (KCNQ1 + KCNE1)_APO_, and (KCNQ1 + KCNE1)_PIP2_ structures, revealing that KCNE1 induces structural changes of the entire ion conduction pathway components including SF, central cavity, and activation gate (Fig. 3e, f). Two major structural rearrangements occurred:

First, the (KCNQ1 + KCNE1)_APO_ structure reveals a closed pore, with the inner gate residue S349 blocking the ion conduction pathway (< 1 Å), whereas the (KCNQ1 + KCNE1)_PIP2_ structure adopts an open conformation (S349 expansion > 3 Å) (Fig. 3f, g). Notably, compared to KCNQ1_APO_, the central cavity and the activation gate of (KCNQ1 + KCNE1)_APO_ exhibit horizontal expansion and a downward shift (Fig. 3f), highlighting KCNE1-induced profound structural remodeling of the pore. For instance, G345, a small-sized amino acid, forms the narrowest point of the central cavity even when the activation gate is in the open state^19,32^ (Fig. 3f). Previously, it was shown that G345 plays a critical role in the partial dehydration of permeating K^+^ ions, thereby regulating single-channel conductance.^19^ In (KCNQ1 + KCNE1)_PIP2_, the distance between two opposite G345 residues is significantly wider than that in KCNQ1_PIP2_ (PDB: 7XNN,^30^ increasing from 10.3 Å to 11.2 Å, Supplementary information, Fig. S10), which may provide a structural basis for enhancement of single-channel conductance by KCNE1.^11,19,46^ Notably, detailed molecular dynamics (MD) simulations should be done to further confirm that the (KCNQ1 + KCNE1)_PIP2_ structure adopts an open conformation.

Second, the SF of K_V_ channels consists of a highly conserved signature sequence (T312/I313/G314/Y315/G316, TIGYG in KCNQ1), where backbone carbonyl oxygen atoms form four evenly distributed K^+^ ion binding sites (s1–s4).^30–34^ Detailed structural analysis revealed that the association of KCNE1 disrupts the s2 K^+^ binding site (Fig. 3h), which strongly suggests that KCNE1 changes the function of SF. Previous studies have demonstrated that KCNE1 significantly reduces the Rb^+^/K^+^ permeability ratio of KCNQ1.^16,18,47,48^ Here we tested tail currents at –60 mV following depolarization to +40 mV while perfusing either 100 mM Rb⁺ or 100 mM K^+^ extracellular solutions. Remarkably, while KCNQ1 alone displayed strong Rb^+^ preference (Rb^+^/K^+^ ratio = 3.0 ± 0.2), KCNQ1 + KCNE1 exhibited reversed ion selectivity (Rb^+^/K^+^ ratio = 0.6 ± 0.1). These results are consistent with previous studies^16,18,47,48^ (Fig. 3i). This changed ion preference and the disrupted s2 site in (KCNQ1 + KCNE1)_PIP2_ are consistent with previous findings that Rb^+^ ions are less favored in s2,^47,49^ which may generate an energy barrier that reduces Rb^+^ permeation. Barium ion (Ba^2+^), a classical potassium channel SF blocker,^15,50^ provides another tool to probe KCNE1-induced functional changes of SF. Here we performed Ba^2+^ blockage experiments and found that 1 mM Ba^2+^ inhibited 72% ± 1% of KCNQ1 currents but only 53% ± 2% of KCNQ1 + KCNE1 currents (Supplementary information, Fig. S11). These functional results, consistent with our structural observations, demonstrate that KCNE1 substantially reprograms SF function.

### VSD transition from intermediate to activated state triggers KCNQ1 + KCNE1 channel opening

The KCNQ1 channel can open when the VSD is in both intermediate and activated states.^11,18,23,30,35–37^ Combining the KCNE1-induced structural changes of both VSD and pore, our findings so far outline the structural basis of how KCNE1 modulates the KCNQ1 channel: the KCNQ1 + KCNE1 channel remains closed when the VSD adopts the intermediate state, and the channel opening is strictly coupled to the VSD transition from the intermediate state to the activated state.^16,18,23,30,36,37^ These findings are supported by VCF results showing that, at 0 mV — the membrane potential under which the cryo-EM structures were determined — the VSD of KCNQ1 + KCNE1 is predominantly in the intermediate state, while the pore remains mainly closed (Fig. 3c).

### KCNE1 induces helix-to-loop transitions in VSD–pore coupling

We next aimed to elucidate how KCNE1-induced structural changes affect VSD–pore coupling. A hand-and-elbow gating mechanism was proposed, in which 2 distinct groups of interactions, from the hand site and the elbow site, respectively, are responsible for the dynamic VSD–pore coupling of KCNQ1^11,16,18,23,30,35–37^ (Supplementary information, Fig. S12a, b). In this model, the S4 and the S4–S5 linker adopt a bent-arm conformation, with intra-subunit interactions at the hand site (between S4–S5 linker and lower S6) important for both IO and AO states, and inter-subunit interactions at the elbow site (between S4/S4–S5 linker and adjacent S5’/S6’) specifically responsible for the AO state (Supplementary information, Fig. S12a, b). Between S4 and S4–S5 linker, there is an “S4 hinge” loop that connects S4 and S4–S5 linker^30–34^ (Supplementary information, Fig. S12a, b).

**Fig. 4:**
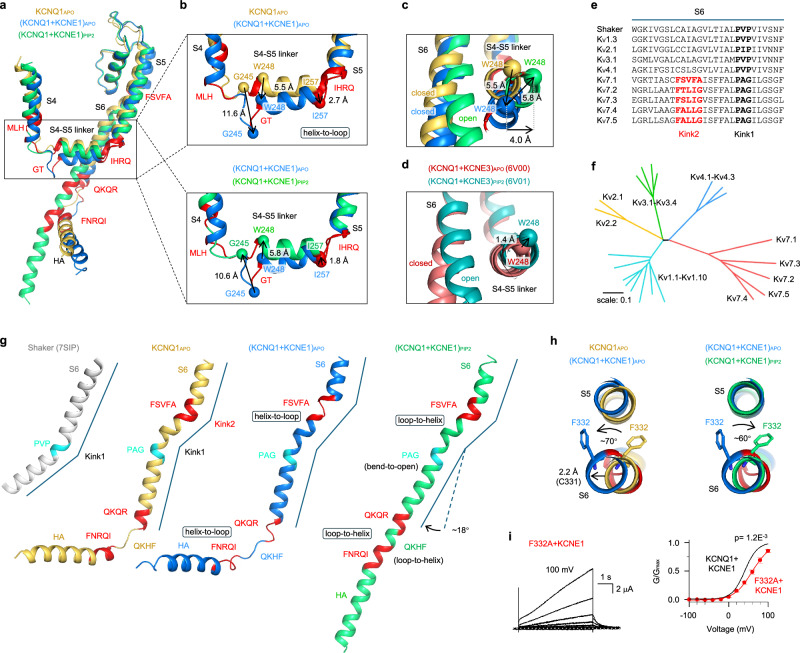
KCNE1 induces helix ↔ loop transitions at the S4–S5 linker and S6, significantly changing the VSD–pore coupling. **a**, **b** Structural comparison of KCNQ1_APO_, (KCNQ1 + KCNE1)_APO_, and (KCNQ1 + KCNE1)_PIP2_ to show three KCNE1-induced helix-to-loop transitions around the S4–S5 linker: M238/L239/H240 (MLH), G246/T247 (GT), and I257/H258/R259/Q260 (IHRQ). These helix-to-loop transitions are preserved during the opening of KCNQ1 + KCNE1. **c** Structural comparison to show that KCNE1 induces a 5.5 Å downward movement of the N-terminus of the S4–S5 linker. During the opening of KCNQ1 + KCNE1, the S4–S5 linker undergoes a 5.8 Å upward movement (with a 4 Å horizontal expansion). **d** The S4–S5 linker of KCNQ1 + KCNE3 undergoes 1.4 Å upward movement with minimum horizontal expansion during opening. **e** S6 sequence alignment of K_V_ channels. The Kink1 motif is conserved across all K_V_s, and Kink2 is conserved in KCNQ channels. **f** Evolutionary tree analysis of all domain-swapped K_V_ channel families (K_V_1–4 and K_V_7). **g** KCNE1 induces three helix-to-loop transitions in S6: one at the Kink2, and the other two at the two ends of the S6/HA linker QKHF (QKQR and FNRQI). These loops undergo loop-to-helix transitions during the opening of S6, following the bend-to-open transition at Kink1. **h** Structural comparison of F332 to show that KCNE1 induces an ~70° rotation (counterclockwise) of the side chain. This rotation is mostly restored during the channel opening. **i** F332A + KCNE1 currents and G–V relation (V_50_ = 59.3 ± 3.0 mV, *n* = 6, *P* = 1.2E-3 with *t*-test).

Remarkably, KCNE1 induced three helix-to-loop transitions around the S4–S5 linker: one at the hand site (I257/H258/R259/Q260, IHRQ motif), disturbing the direct helix connection between S4–S5 linker and S5, and two at the elbow site (M238/L239/H240, MLH and G246/T247, GT motifs), extending the S4 hinge from both ends. Consequently, these helix-to-loop transitions cause substantial downward movement of the S4–S5 linker (5.5 Å at N-terminus W248; 2.7 Å at C-terminus I257, Fig. 4a, b and Supplementary information, Fig. S13a–d). The S4 hinge dropped by an even larger distance (11.6 Å at G245) following the VSD transition from the activated state of KCNQ1_APO_ to the intermediate state of (KCNQ1 + KCNE1)_APO_ (Fig. 4a, b and Supplementary information, Fig. S13a–d and Video S2). As an upward movement of the S4–S5 linker is required for the voltage-dependent opening, these pronounced downward movements of S4–S5 linker and S4 hinge, as well as the flexible loop connections, would likely create an energy barrier that stabilizes pore closure.

To elucidate the VSD–pore coupling that supports the KCNQ1 + KCNE1 channel opening, we performed a structural comparison of (KCNQ1 + KCNE1)_APO_ and (KCNQ1 + KCNE1)_PIP2_. Following the VSD transition from intermediate to activated state, the elbow site undergoes a large upward movement (10.6 Å for Q244, and 5.8 Å for W248), while the hand site moves up by only 1.8 Å (at I257) (Fig. 4a, b). Notably, from the top view, the N-terminus of S4–S5 linker undergoes a 4 Å horizontal expansion, driving the pore opening with a diagonal upward movement (Fig. 4c and Supplementary information, Fig. S14). This gating motion at S4–S5 linker differs fundamentally from those of both KCNQ1 + KCNE3 and KCNQ1, which either shows only 1.4 Å upward movement with negligible horizontal expansion (Fig. 4d), or ~6 Å upward movement but < 1 Å horizontal expansion (Supplementary information, Fig. S14). These differential S4–S5 linker motions during gating provide a structural perspective that KCNQ1 and KCNQ1 + KCNE3 channels favor IO state, while KCNQ1 + KCNE1 only opens in AO state.^11,16,18,23,30,35–37^

### KCNE1 induces helix-to-loop transitions in S6 and HA

Unlike other K_V_ channels (e.g., the Shaker channel) feature a single conserved PVP hinge sequence (P343/A344/G345, PAG in KCNQ1^51^) in the mid-S6 to form a kink and enable a bend-to-open process (Fig. 4e, g), KCNQ channels (KCNQ1–5) exhibit a distinctive dual-hinge architecture with an additional kink-forming sequence (F332/S333/V334/F335/A336, FSVFA in KCNQ1) above PAG, creating two kinks in S6 that diversify the gating (Fig. 4e, g). In line with this observation, we performed an evolutionary analysis of K_V_ channels with domain-swapped architecture (K_V_1–4, and K_V_7), and found that clades of K_V_7 channels show a significantly higher degree of evolutionary divergence than K_V_1–4 (Fig. 4f).

Structural comparison of KCNQ1_APO_ and (KCNQ1 + KCNE1)_APO_ revealed that KCNE1 induces another three major conformational changes: (1) an ~70° counterclockwise rotation of the conserved F332 side chain (Fig. 4h); (2) as a result, the FSVFA motif undergoes a helix-to-loop unfolding (Fig. 4g and Supplementary information, Fig. S13e–h); (3) two more helix-to-loop transitions at both ends of the S6–HA linker (Q357/K358/Q359/R360, QKQR and F364/N365/R366/Q367/I368, FNRQI) that elongate the linker from R360/Q361/K362/H363/F364, RQKHF to QKQRQKHFNRQI (Fig. 4g and Supplementary information, Fig. S13e–h).

The F332 residue is well conserved among KCNQ channels (Fig. 4e). Physically, it points to the S5 and undergoes an ~10° clockwise rotation during KCNQ1 opening (top view of KCNQ1_APO_ and KCNQ1_PIP2_ (PDB: 7XNN^30^); Supplementary information, Fig. S15). The association of KCNE1, however, counterclockwisely rotates F332 by ~70° to the other side of S5, which expands the helix above F332 (2.2 Å for C331, Fig. 4h), and breaks hydrogen bonds below F332 (shrinking V334 by 2.9 Å; Supplementary information, Fig. S16), inducing a helix-to-loop unfolding of the FSVFA motif (Fig. 4g, h). Notably, this helix-to-loop unfolding induced an upward movement of S6 above the FSVFA motif (e.g., 1.4 Å for K326; Supplementary information, Fig. S16), and a downward shift of the S6 and S5 segments (2.3 Å for R259 in S5 and 3.0 Å for K354 in S6; Supplementary information, Fig. S16). On top of these changes in S6, KCNE1 induced two additional helix-to-loop transitions that elongate the S6–HA linker (Fig. 4g). Following these changes, the entire cytosolic domain has a ~6 Å downward movement (Figs. 1d and 4g). These profound secondary structural changes may also contribute to establishing a higher energy barrier that stabilizes the channel in the closed state.

During the opening of KCNQ1 + KCNE1, F332 rotates backward by ~60° (clockwise, Fig. 4h). Meanwhile, the FSVFA motif and the QKQRQKHFNRQI loop refold to helical conformations, enabling S6 and HA to form a continuous helix, and the cytosolic domains HA/HB together with CaM undergo ~18° rotation (Fig. 4g). Concurrently, the PAG kink mediates a bend-to-open transition in S6 that facilitates channel opening (Fig. 4g). Furthermore, we found that F332A + KCNE1 showed a significantly right-shifted G–V relation (V_50_ = 62.6 ± 3.7 mV, Fig. 4i), which confirms the importance of F332 rotation during KCNQ1 + KCNE1 gating.

### A unique dual-PIP2 modulation mechanism of KCNQ1 + KCNE1

The membrane phospholipid PIP2 is essential for both KCNQ1 and KCNQ1 + KCNE1 channel gating, particularly in mediating VSD–pore coupling.^52^ Although structural studies have identified a canonical PIP2-binding site, involving basic residues from the S2–S3 linker, S4–S5 linker, and CaM,^30,32^ two main questions remain unresolved. Firstly, current structures (both KCNQ1 and KCNQ1 + KCNE3) show only one PIP2 binding per subunit, and PIP2 binding appears strictly limited to the activated state VSD,^30–34^ leaving it unclear whether additional PIP2 molecules participate in channel gating and whether PIP2 binding can occur in VSD in other than the activated state. Secondly, KCNE1 enhances PIP2 sensitivity of KCNQ1 ( ~100-fold).^16,36,53^ However, the structural basis for this dramatically enhanced PIP2 sensitivity remains unknown.

**Fig. 5:**
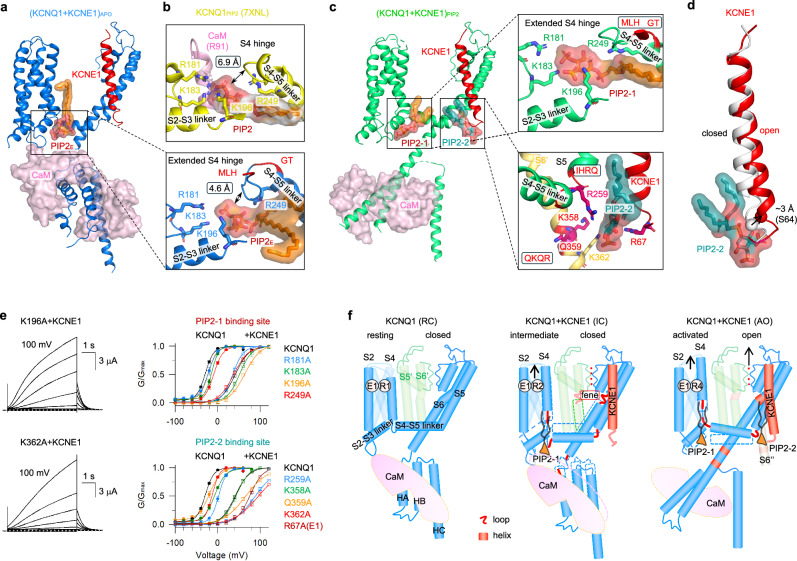
A unique dual-PIP2 modulation mechanism of KCNQ1 + KCNE1. **a** In (KCNQ1 + KCNE1)_APO_, an endogenous PIP2_E_ molecule was observed in the canonical PIP2-binding site (involving residues R181, K183, K196, and R249), while the CaM is in the attached mode. **b** The extended S4 hinge in (KCNQ1 + KCNE1)_APO_ shows smaller distance to the PIP2 molecule than that in KCNQ1_PIP2_ (from 4.6 Å to 6.9 Å, PDB: 7XNL^30^). **c** In (KCNQ1 + KCNE1)_PIP2_, two PIP2 molecules (PIP2-1 and PIP2-2) were observed in each KCNQ1 subunit: PIP2-1 in the canonical site, and PIP2-2 between S4–S5 linker, S6’, and KCNE1 (involving residues R259, K358, Q359, K362, and KCNE1-R67), while the CaM is in the detached mode. Density maps and models of PIP2-1 and PIP2-2 are shown in orange and dark green, respectively. **d** Structural comparison of KCNE1 between (KCNQ1 + KCNE1)_APO_ (gray) and (KCNQ1 + KCNE1)_PIP2_ (red) to show that PIP2-2 binding induces ~3 Å movement of the bottom part of KCNE1 without altering the top part. **e** G–V relations of alanine mutagenesis scanning of PIP2-1 and PIP2-2 binding residues in the absence and presence of KCNE1: V_50_ = –22.4 ± 2.8 mV for R181A (*n* = 4); V_50_ = 36.1 ± 2.4 mV for R181A + KCNE1 (*n* = 5); V_50_ = –15.1 ± 1.5 mV for K183A (*n* = 4); V_50_ = 52.2 ± 3.5 mV for K183A + KCNE1 (*n* = 4); V_50_ = –20.8 ± 1.0 mV for K196A (*n* = 4); V_50_ = 63.8 ± 3.4 mV for K196A + KCNE1 (*n* = 4); V_50_ = –9.3 ± 1.6 mV for R249A (*n* = 5); V_50_ = 37.9 ± 2.7 mV for R249A + KCNE1 (*n* = 4); V_50_ = 2.4 ± 1.2 mV for R259A (*n* = 6); V_50_ = 86.0 ± 5.2 mV for R259A + KCNE1 (*n* = 5); V_50_ = –6.9 ± 0.9 mV for K358A (*n* = 4); V_50_ = 43.8 ± 3.2 mV for K358A + KCNE1 (*n* = 6); V_50_ = –19.6 ± 2.5 mV for Q359A (*n* = 4); V_50_ = 66.1 ± 4.8 mV for Q359A + KCNE1 (*n* = 5); V_50_ = –14.4 ± 0.9 mV for K362A (*n* = 4); V_50_ = 95.8 ± 9.4 mV for K362A + KCNE1 (*n* = 5). For R67 on KCNE1, KCNQ1 + R67A was tested (V_50_ = 76.0 ± 1.2 mV, *n* = 4). **f** Cartoon schemes showing KCNE1-induced global structural remodeling of KCNQ1, and the gating process of KCNQ1 + KCNE1. Briefly, KCNE1 stabilizes the VSD in the intermediate state (with a PIP2 binds to the canonical site), and induces six helix-to-loop transitions (red lines) per KCNQ1 subunit, keeping the KCNQ1 + KCNE1 channel in the IC state. Upon depolarization, the VSD transitions to the activated state, three loops from S6 undergo loop-to-helix transitions to form a continues helix along S6 and HA, and a second PIP2 binds to stabilize the KCNQ1 + KCNE1 channel in the AO state.

Resolution of the (KCNQ1 + KCNE1)_APO_ structure, prepared without adding exogenous PIP2, unexpectedly revealed well-defined PIP2 density at the canonical site (Fig. 5a), which we attribute to endogenous PIP2 retention surviving protein purification: Firstly, the KCNE1 induces ~10° counterclockwise VSD rotation, along with 6–8 Å displacements in the S2–S3 linker, S4–S5 linker, and the entire cytosolic domain may better “clamp” the head group of PIP2 (Fig. 5a and Supplementary information, Fig. S17a, b); Secondly, the extended S4 hinge undergoes a pronounced downward shift, positioning it closer to the neck and tail regions of PIP2 (from 6.9 Å to 4.6 Å, Fig. 5a, b), which stabilizes PIP2 binding. These results not only elucidate KCNE1’s role in boosting PIP2 sensitivity and preserving endogenous PIP2 during protein preparation but also demonstrate that (KCNQ1 + KCNE1)_APO_ adopts a well-coupled structure that binds PIP2 in the intermediate state VSD.

To uncover the PIP2 modulation mechanism, we next prepared (KCNQ1 + KCNE1)_EM_ samples in the presence of exogenous PIP2. Interestingly, in the (KCNQ1 + KCNE1)_PIP2_ structure, we identified two distinct PIP2 molecules (PIP2-1 and PIP2-2) per subunit: PIP2-1 occupies the canonical site, coordinated by R181, K183, K196, and R249 (Fig. 5c), while PIP2-2 binds in a new dynamic pocket formed by the S4–S5 linker (R259), adjacent S6’ (K358, Q359, and K362), and the C-terminus of KCNE1 (R67) (Fig. 5c and Supplementary information, Fig. S17c, d). These two PIP2 molecules together contribute to the channel gating: While PIP2-1 remains stably bound throughout the gating process, PIP2-2 exhibits AO state-dependent binding, where involved binding residues move away from each other in (KCNQ1 + KCNE1)_APO_ (Supplementary information, Fig. S17e). Notably, the PIP2-2 binding stabilizes the lower region of KCNE1, enabling clear resolution of residues R67 (directly participates in the PIP2-2 binding) and S68 in (KCNQ1 + KCNE1)_PIP2_, which was missing in (KCNQ1 + KCNE1)_APO_ (Fig. 5a–c). Within the S6’, PIP2-2 interacts with residues K358, Q359, and K362, a region that undergoes the characteristic loop-to-helix transition during channel opening (Fig. 5c). Furthermore, PIP2-2 binding also triggers two distinct structural rearrangements in KCNE1: (1) a loop-to-helix transition at the I61/M62/L63 (IML) motif, and (2) an ~3 Å displacement of KCNE1’s lower region without altering its upper portion (Fig. 5d). These observations collectively highlight the dynamic nature of PIP2-2 binding and the essential mechanistic role of helix↔loop transitions during channel gating.

Previous studies have demonstrated that mutating PIP2-binding residues can result in right-shifted G–V relations.^30,52^ To validate the PIP2-1 and PIP2-2 binding sites, we performed alanine scanning mutagenesis on key interacting residues. Mutations in PIP2-1 binding residues caused moderate right-shifts in G–V relations (ΔV range of 7.7–20.2 mV, Fig. 5e), which is consistent with previous studies.^30,52^ When co-expressed with KCNE1, the mutant I_Ks_ channels show similarly right-shifted G–V relations (ΔV range of –5.7–22.5 mV, Fig. 5e). In contrast, PIP2-2 mutations caused more pronounced right-shifts (ΔV range of 10.3–31.7 mV for KCNQ1 alone, and ΔV range of 2.3–53.9 mV when co-expressed with KCNE1, Fig. 5e). To further confirm these PIP2-binding residues, we also performed current rundown experiments: both K196A + KCNE1 and Q359A + KCNE1 channels exhibited significantly faster rundown compared to WT I_Ks_ (Supplementary information, Fig. S18), consistent with previous reports on PIP2-interacting residues.^52^

These findings confirm the key roles of both PIP2 in channel activation: both (KCNQ1 + KCNE1)_APO_ (closed pore) and (KCNQ1 + KCNE1)_PIP2_ (open pore) structures have canonical PIP2 binding, indicating that PIP2-1 maintains stable binding at the canonical site throughout channel gating, while only the (KCNQ1 + KCNE1)_PIP2_ structure exhibits a second PIP2 binding, suggesting that the PIP2-2 only binds when the channel is open and dissociates when the channel is closed. This also suggests that the binding of PIP2-2 facilitates pore opening. Notably, comprehensive PIP2 dose-response analyses of both WT and mutant I_Ks_ channels remain essential to further experimentally validate the proposed PIP2-binding residues.

In summary, our study deciphers how the KCNE1-induced global structural remodeling is coupled to the comprehensive functional modulations of KCNQ1 (Fig. 5f). KCNE1 occupies a strategic position at the interface of three KCNQ1 subunits, inducing: (1) an ~10° counterclockwise VSD rotation that stabilizes the intermediate state and PIP2-1 binding (Supplementary information, Video S1); (2) three helix-to-loop transitions around the S4–S5 linker that lower both sides of the S4–S5 linker (Supplementaryinformation, Video S2); (3) another three helix-to-loop transitions in S6 and HA that shift the pore and cytosolic domains downward, stabilizing the closed conformation in the pore (Supplementary information, Video S3). Notably, a fenestration (fene, Fig. 5f) site is induced by these secondary structure transition, which will be investigated in the following session. During KCNQ1 + KCNE1 activation, VSD transition to the activated state diagonally elevates the S4–S5 linker, creating a second PIP2-binding site that bridges the S4–S5 linker, KCNE1, and S6’. This process triggers three loop-to-helix transitions in S6 and HA, forming a continuous helix, while the PAG kink undergoes the bend-to-open transition to open the channel in AO state (Supplementary information, Videos S4–S6).

### The helix ↔ loop transitions create a fenestration in KCNQ1 + KCNE1

**Fig. 6:**
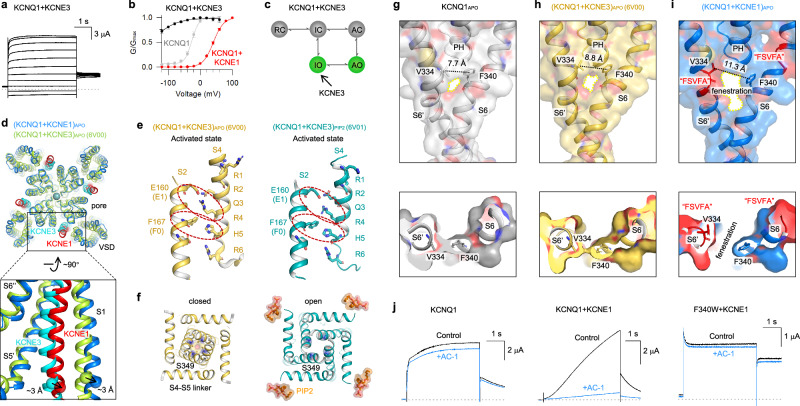
The KCNE1-induced helix ↔ loop transitions create a fenestration in the I_Ks_ channel. **a**, **b** Activation currents and G–V relation of KCNQ1 + KCNE3. G–V relations of KCNQ1 (gray) and KCNQ1 + KCNE1 (red) are also shown. **c** Cartoon scheme showing that KCNE3 enhances IO state. **d** Structural comparison of (KCNQ1 + KCNE1)_APO_ and (KCNQ1 + KCNE3)_APO_ (PDB: 6V00^32^) to show that KCNE1 induces further ~3 Å movement of S1 compared to KCNE3. **e** Both the VSDs of (KCNQ1 + KCNE3)_APO_ (PDB: 6V00) and (KCNQ1 + KCNE3)_PIP2_ (PDB: 6V01^32^) are in activated state. Red circles highlight E160/R237 and F167/H240 interactions. **f** Structural comparison of the activation gate (S349) between (KCNQ1 + KCNE3)_APO_ (PDB: 6V00) and (KCNQ1 + KCNE3)_PIP2_ (PDB: 6V01^32^). **g** In KCNQ1_APO_, adjacent S6 segments remain tightly packed (V334 ↔ F340 distance is 7.7 Å). **h** In (KCNQ1 + KCNE3)_APO_, no helix-to-loop transition was observed, and adjacent S6 segments remain tightly packed (V334 ↔ F340 distance is 8.8 Å). **i** In (KCNQ1 + KCNE1)_APO_, KCNE1-induced helix-to-loop transitions at the FSVFA motifs force S6 helices to separate, creating a fenestration pocket (V334 ↔ F340 distance is 11.3 Å). **j** Representative activation currents of KCNQ1, KCNQ1 + KCNE1, and F340W + KCNE1 before and after adding 1 μM AC-1.^29^ To avoid potential inactivation at high voltage, the F340W + KCNE1 channel currents were recorded at 0 mV.^51^

KCNE3, another epithelial-specific auxiliary subunit, enables the KCNQ1 + KCNE3 channel to remain constitutively open across physiological voltages, helping maintain the resting membrane potential required for Cl⁻ secretion in the gut^8,10,13,35,38,39^ (Fig. 6a). Our prior work demonstrated that KCNE3 markedly stabilizes the IO state, resulting in a strongly left-shifted G–V relationship^35^ (Fig. 6b, c). These “opposite effects” of KCNE1 and KCNE3 on KCNQ1 strongly suggest that the KCNQ1 + KCNE1 and KCNQ1 + KCNE3 channels have fundamentally distinct structures.

We next compared the structures of the KCNQ1 + KCNE1 and KCNQ1 + KCNE3 channels, identifying three major differences: (1) VSD displacement: the KCNE1-induced movement of the VSD is more pronounced than that observed with KCNE3, with the VSD (S1) exhibiting an additional ~3 Å displacement compared to that in the KCNQ1 + KCNE3 structure (PDB: 6V00,^32^ Fig. 6d); (2) VSD activation state: while (KCNQ1 + KCNE1)_APO_ and (KCNQ1 + KCNE1)_PIP2_ exhibit intermediate and activated states of VSD, respectively (Fig. 3a), both (KCNQ1 + KCNE3)_APO_ and (KCNQ1 + KCNE3)_PIP2_ structures show E160/R237 and F167/H240 interactions, stabilizing the VSDs in the activated state^32^ (Fig. 6e); (3) PIP2 binding mechanism: unlike KCNQ1 + KCNE1 utilizing a dual-PIP2 modulation mechanism (Fig. 5), KCNQ1 + KCNE3 relies on a single PIP2 molecule bound at the canonical site for channel opening (Fig. 6f). Collectively, our structural analyses provide high-resolution insights into the distinct regulatory mechanisms of KCNE1 and KCNE3.

KCNE1-induced global secondary structural transitions also reshape the pharmacology of KCNQ1 and KCNQ1 + KCNE1 channels. In the KCNQ1_APO_ and (KCNQ1 + KCNE3)_APO_ (PDB: 6V00^32^) structures, adjacent S6 segments remain tightly packed, sealing the central cavity (V334 ↔ F340 distances are 7.7 Å and 8.8 Å, respectively; Fig. 6g, h). However, in (KCNQ1 + KCNE1)_APO_, the KCNE1-induced helix-to-loop transition at the FSVFA motif forces S6 helices to separate, creating a distinct fenestration pocket (V334 ↔ F340 distance is 11.3 Å, Fig. 6i). This KCNE1-dependent fenestration provides a highly selective pocket for developing KCNQ1 + KCNE1 modulators. Consistent with this observation, the Seebohm lab has previously identified AC-1, a highly selective and potent blocker of KCNQ1 + KCNE1 with no activity against KCNQ1 alone^29^ (Fig. 6j). Their functional studies demonstrated that KCNE1 binding opens fenestrations between S6 helices, enabling AC-1 binding.^29^ Our structural analysis of (KCNQ1 + KCNE1)_APO_ identifies F340 as a critical residue lining the fenestration pocket (Fig. 6i). Supporting this, we found that the F340W + KCNE1 mutant abolishes AC-1 sensitivity (Fig. 6j), indicating that the bulky tryptophan side chain sterically blocks the fenestration, preventing AC-1 binding.

## Discussion

The distinctly slow activation of the KCNQ1 + KCNE1 channel is a defining feature of its physiological function in cardiac repolarization. While extensive functional studies have probed how KCNE1 modulates KCNQ1,^8,10–14,16,24,29,35,40,44,54–57^ our structural analysis provides mechanistic insights that unify and extend these observations: (1) KCNE1 stabilizes the VSD in the intermediate state, slowing down both the transition to the activated state (during activation) and the return to the resting state (during deactivation). (2) KCNE1 induces three helix-to-loop transitions around the S4–S5 linker, slowing down the VSD–pore coupling process to open the channel. The downward displacement of the S4–S5 linker may also raise the energy barrier, further stabilizing the intermediate state VSD and closed pore. (3) KCNE1 promotes another three helix-to-loop transitions in S6 and HA, increasing pore flexibility and energetically disfavoring the bend-to-open transition required for channel opening. Consistent with these structural observations, functional data showed that the pore opening of KCNQ1 + KCNE1 follows the second (slow) component of VSD activation.^16,35,44^ (4) Dual PIP2-mediated modulation, especially the newly discovered PIP2-2 that bridges S4–S5 linker, KCNE1 and S6’. These structural determinants undergo time-consuming helix ↔ loop transitions during gating, which may also lead to delayed pore opening. (5) A large structural rearrangement at the entire cytosolic domain, including the ~18° rotation and ~6 Å downward movement at HA/HB/CaM during the gating process, also slows the activation and deactivation processes.

Secondary structure transitions during ion channel gating have been previously identified across diverse channel families. For example, in non-domain-swapped HCN1, channel opening converts the S4–S5 linker from a short loop to an extended flexible loop.^58^ The non-domain-swapped hSlo1 + β2_N_-β4 complex in the intermediate state reveals a helix-to-loop transition at its hinge glycine of S6.^59^ The domain-swapped Na_V_1.8 undergoes a helix-to-loop transition in DIII-S6, similarly centered at the glycine hinge.^60^ Another loop-to-helix transition at the hinge between S4–S5 linker and S5 was induced by the M11 mutations in Na_V_1.7.^61^ In ligand-gated TRPM8 channel, the C-terminus of S6 undergoes a loop-to-helix transition during channel opening.^62^ Here, we demonstrate that an auxiliary subunit of a K_V_ channel with domain-swapped architecture leverages large-scale helix-loop transitions to profoundly reprogram channel function and pharmacology. The conservation of secondary structure transitions across evolutionarily distant channels, from non-domain-swapped K channels to domain-swapped K_V_, Na_V_, and ligand-gated TRP channels, strongly suggests that these transitions represent a fundamental mechanism of ion channel gating. Beyond primary structure (sequence) and static secondary structure (α-helix, β-sheet, flexible loop, etc.) of ion channel proteins, these dynamic helix ↔ loop switches introduce a new layer of structural control that fine-tunes ion channel function.

The PIP2 modulation mechanism remains a central focus in studies of KCNQ channels,^30,34,52,63,64^ with functional studies identifying multiple potential binding sites beyond the canonical site, including the S4–S5/S6 C-termini and HA-HC regions.^53,63,65–69^ Unlike previous structures of KCNQ1 and KCNQ1 + KCNE3 showing that PIP2 only binds to activated state VSD with open pore conformation,^30–32,34^ our study reveals a novel dual-PIP2 modulation mechanism specific to KCNQ1 + KCNE1: Mandala and MacKinnon reported that the VSD state regulates PIP2 accessibility, with the “up” state favoring and the “down” state occluding PIP2 binding.^34^ Here, we found that PIP2 can bind at the canonical site in the intermediate state VSD. Since KCNE1 strongly stabilizes the intermediate state VSD (V_50_ of F_1_ < –120 mV), the VSD rarely returns to the resting state under physiological voltages, permitting continuous PIP2-1 binding during channel gating. For the second PIP2-2 binding, all residues coordinating this PIP2 molecule are located at flexible loop regions that undergo substantial conformational rearrangements during gating. This reveals a new mode of dynamic modulation by PIP2. Therefore, our results provide a high spatial and temporal resolution in PIP2 binding and modulation of KCNQ1 channels. Future studies, including comprehensive PIP2 dose-response analyses and MD simulations, will be valuable to further investigate the dynamics of PIP2 binding and modulation.

Importantly, our high-resolution structures of KCNQ1 + KCNE1 channels in both closed and open conformations provide critical insights for structure-based drug discovery in LQTS. Our results revealed several unique and druggable pockets. (1) The fenestration site. The KCNE1-induced fenestration is absent in KCNQ1 and KCNQ1 + KCNE3, providing a structural basis for developing highly specific KCNQ1 + KCNE1 modulators that may reduce side effects in non-cardiac tissues. Furthermore, this is a pore-embedded binding pocket. Compounds targeting this site would bypass dysfunctional pathogenic mutations that impair VSD activation and VSD–pore coupling. (2) The PC binding pocket. Our findings confirm the critical role of this conserved lipid-binding site. Studies from the Larsson lab and the Liin lab have shown that this pocket mediates the binding of polyunsaturated fatty acids, and can be effectively modulated to rescue the dysfunction of LQT1 mutations.^25–27^ (3) The dual PIP2-binding site. Besides the canonical PIP2-binding site, we identified a new PIP2-binding site that can bind PIP2 when the channel is open and dissociate PIP2 when the channel is closed. Small molecules that bind at these sites may stabilize the channel in the open conformation. Studies from the Cui lab show that a PIP2 substitute rescues defective I_Ks_ currents when endogenous PIP2 is depleted.^70^ (4) The remodeled elbow pocket. ML277 is a KCNQ1-specific activator that was found to bind at the elbow pocket.^12,30^ While KCNE1 disrupts the ML277-binding site, its extensive restructuring of this VSD–pore coupling interface offers new opportunities for developing selective modulators. Together, these structural insights advance our understanding of the unique gating properties of the KCNQ1 + KCNE1 channel and highlight several promising avenues for the development of next-generation therapeutics for LQTS.

## Materials and methods

### Constructs and mutagenesis

Overlap extension and high-fidelity PCR were used for making each KCNQ1 channel point mutation, which was confirmed by DNA sequencing. The cRNAs of WT KCNQ1 and all mutants were synthesized using the mMessage T7 polymerase kit (Applied Biosystems-Thermo Fisher Scientific) for oocyte injections. RNAs were kept at –80 °C.

For structural analysis, we generated the constructs with the full-length KCNE1 sequence and a linker^40^ inserted at the N-terminus of the truncated constructs of KCNQ1 (residues 76–620). *Eco*RI and *Not*I sites were used for cloning the (KCNQ1 + KCNE1)_EM_ construct into the pEGBacMam expression vector with a C-terminal Maltose Binding Protein (MBP)-10× His tag. The human CaM gene was cloned into BacMam expression vector without any tags. (KCNQ1 + KCNE1)_EM_ and CaM complex were heterologously expressed in Human Embryonic Kidney (HEK) 293 F cells. When cell density reached 2.0–3.0 × 10^6^ cells/mL, the cells were cotransfected with plasmids at mass ratio of 10:1 for (KCNQ1 + KCNE1)_EM_ and CaM. For 1-L HEK 293 F cell culture, the plasmids (~1 mg) were premixed with linear polyethyleneimines (PEIs) (MKbio) in 50-mL fresh medium for 15–30 min. The mixture was then added into cell culture followed by 15-min incubation. After 24 h incubation at 37 °C, 10 mM sodium butyrate was added to induce the protein expression at 30 °C. Cells were harvested after 48 h, then flash-frozen in liquid nitrogen and stored at −80 °C until needed.

Cell pellets were resuspended in hypotonic buffer (20 mM Tris-HCl, pH 8.0, 20 mM KCl, 0.5 mM MgCl_2_, 2 mM DTT) supplemented with a protease inhibitor cocktail (Selleck) for 40 min with gentle agitation. Crude cell membranes were collected by ultra-centrifugation at 105,400× *g* for 45 min. The membranes were then re-suspended and solubilized in buffer containing 20 mM Tris-HCl, pH 8.0, 150 mM KCl, 2 mM DTT, 0.5% LMNG:CHS (10:1, w/w) for 2–2.5 h at 4 °C. After centrifugation at 105,400× *g* for 45 min, the supernatant was incubated with amylose resin (NEB) at 4 °C for 2 h. Detergent was exchanged on resin by a series of washing steps in 20 mM Tris-HCl, pH 8.0, 150 mM KCl, 2 mM DTT supplemented with different detergents: first 0.1% LMNG, 0.01% CHS, 0.1% GDN, then 0.1% GDN and 0.05% GDN for 20 column volumes each. The protein was eluted with 40 mM Maltose in wash buffer, and subsequently concentrated by a 100-kDa concentrator (Millipore) before being injected onto a Superose 6 Column (GE Healthcare) equilibrated with 20 mM Tris-HCl, pH 8.0, 150 mM KCl, 2 mM DTT, 0.03% GDN. The peak fractions were pooled, concentrated to 4–5 mg/mL using a 100-kDa MWCO centrifugal device (Millipore) before cryo-EM sample preparation. For the (KCNQ1 + KCNE1)_PIP2_ sample, the purified protein was incubated with 1 mM PIP2 (diC8-PIP2, Echelon) for 30 min.

### Cryo-EM sample preparation and data acquisition

For grids preparation, 2.5–3.0 μL of concentrated protein complex was loaded onto glow-discharged holey carbon grids (Quantifoil Au R1.2/1.3, 300 mesh) at 4 °C under 100% humidity. Grids were blotted for 3.5 s and plunge-frozen in liquid ethane using a Vitrobot Mark IV (FEI). Micrographs were acquired on a Titan Krios microscope (FEI) operating at a voltage of 300 kV. Summary of detailed data collection was shown in Supplementary information, Table S1.

### Cryo-EM data processing

Images of all datasets were imported into cryoSPARC v4.1.1. After motion correction, electron-dose weighting and CTF estimation, the initial particles were picked by cryoSPARC blob picker and 2D classification was processed to generate a template. For (KCNQ1 + KCNE1)_APO_ datasets, after two rounds of 2D classification, the good particles proceeded to Ab-initio reconstruction and heterogeneous refinement. Then we used the best class of particles to generate a template. And the particles were picked by cryoSPARC template picker. Auto-picked particles were visually examined to remove false positives and were further cleaned up by multiple rounds of 2D classification. The good particles proceeded to two rounds of Ab-initio reconstruction and heterogeneous refinement. The final particle sets were re-extracted with original box size and further applied for final nonuniform refinement and local refinement. For (KCNQ1 + KCNE1)_APO_ datasets, we merged the particles of the two best classes to remove duplicates and the final particle sets were re-extracted with original box size and further applied for final nonuniform refinement and local refinement.

### Model building and refinement for cryo-EM structures

Auto-sharpen of the Phenix program was used for map sharpening. The reference models (PDB: 6UZZ, 6V00, and 6V01) were rigid-body fitted into the EM density map using ChimeraX. The fit was further adjusted using the jiggle fit function in Coot. Further manual adjustment with the real-space refine zone function in Coot was used to generate an atomic model. The generated model was further refined using the real_space_refine tool in Phenix. To enhance the accuracy and reproducibility of the low-resolution structural model (3.4 Å), we adopted a comprehensive strategy of template-assisted modeling + de novo fitting + iterative refinement. The specific process and validation indicators are as follows: (1) using the core domain of the homologous protein (PDB: 6V01, resolution 3.9 Å) as the template, the ChimeraX “align and fit” function was used to initially position the model onto the cryo-EM density map; (2) for flexible regions (such as the N-terminal loop region and the interface between subunits), de novo fitting was performed using Coot 0.9.8, optimizing the matching degree of side chains and density residue by residue; (3) Ramachandran plot statistics: 94% of the residues were located in the optimal region, and the MolProbity score was < 2.0, indicating that the geometric structure of the model is reasonable.

MolProbity and Mtriage were used for validation. The pore radii were calculated using HOLE. PyMOL3.1.3 and ChimeraX were used to further analyze the structure and generate figures.

### Oocyte preparation and ion channel expression

Mature oocytes (at stage V or VI) were obtained from *Xenopus laevis* by laparotomy, following the protocol approved by the Animal Studies Committee of Macau University of Science and Technology (Protocol#: MUST-NSFC-2021022601HPP). Collagenase (Sigma Aldrich) at 0.5 mg/mL concentration was used to digest oocytes. For cRNA micro-injection, WT or mutant KCNQ1 cRNAs (9.2 ng) with or without KCNE1 cRNA were injected into each oocyte with a 4:1 (KCNQ1:KCNE1) weight ratio. This allows a saturate KCNE1 association with KCNQ1.^18,71^ Injected cells were incubated in ND96 solution: 96 mM NaCl, 2 mM KCl, 1.8 mM CaCl_2_, 1 mM MgCl_2_, 5 mM HEPES, 2.5 mM CH_3_COCO_2_Na, 1:100 Pen-Strep, pH 7.6) at 18 °C for 2–6 days for electrophysiology recordings.

### Two-electrode voltage clamp (TEVC) and VCF

Microelectrodes (Sutter Instrument) were made with a Sutter (P-1000) puller with 1–3 mΩ resistances when filled with 3 M KCl. The extracellular solution was ND96 solution without CH_3_COCO_2_Na. Currents were recorded with an OC-725D TEVC amplifier (Warner). Currents were sampled at 1 kHz and low-pass-filtered at 2 kHz. For VCF experiments, oocytes were incubated on ice for 30 min in labeling solution: 10 μM Alexa 488 C5-maleimide (Molecular Probes) in 100 mM K^+^ solution. After labeling, oocytes were washed three times with the ND96 solution before VCF recordings. All recordings were performed at room temperature of 20–22 °C.

### Electrophysiology data analysis

Data were analyzed with Clampfit (Axon Instruments), Sigmaplot (SPSS), and IGOR (Wavemetrics). Due to photo-bleaching, fluorescence signals were baseline subtracted. G–V and F–V curves were fitted with single or double Boltzmann equations in the form of 1/(1 + exp(−z × *F* × (*V* − *V*_*1/2*_)/*RT*)), where *V* is the voltage, z is the equivalent valence, *V*_*1/2*_ is the half-maximal voltage, *F* is the Faraday constant, *R* is the gas constant, and *T* is the absolute temperature. For double mutant cycle analysis, the activation energy was measured by ΔG = –*z* × *F* × *V*_*1/2*_. *F* is the Faraday constant. Both *z* and *V*_*1/2*_ were estimated by fitting G–V relations of each channel with a single Boltzmann equation.

### Statistical analysis

Averaged data were presented as mean ± standard error of mean (SEM) with *n* specifying the number of independent experiments. Statistical analyses (*t*-test, paired *t*-test, one-way ANOVA and post-hoc mean comparison Tukey test or Dunnett test) were performed with Sigmaplot (SPSS) and R software (4.1.2 version, multcomp package). Statistical significance was set as “*” *P* < 0.05, “**” *P* < 0.01, and “***” *P* < 0.001.

## Supplementary information

Supplementary Video S1

Supplementary Video S2

Supplementary Video S3

Supplementary Video S4

Supplementary Video S5

Supplementary Video S6

Supplementary Figure S1

Supplementary Figure S2

Supplementary Figure S3

Supplementary Figure S4

Supplementary Figure S5

Supplementary Figure S6

Supplementary Figure S7

Supplementary Figure S8

Supplementary Figure S9

Supplementary Figure S10

Supplementary Figure S11

Supplementary Figure S12

Supplementary Figure S13

Supplementary Figure S14

Supplementary Figure S15

Supplementary Figure S16

Supplementary Figure S17

Supplementary Figure S18

Supplementary Table S1
