# Supplementary material for: Secondary structure transitions and dual PIP2 binding define cardiac KCNQ1-KCNE1 channel gating
Source: Cell Res. 2025 Oct 2;35(11):887–99. doi: 10.1038/s41422-025-01182-9 (PMC12589563; doi:10.1038/s41422-025-01182-9)
Supplement: Supplementary file 7 — Supplementary Figure S1 [file 41422_2025_1182_MOESM7_ESM.pdf]

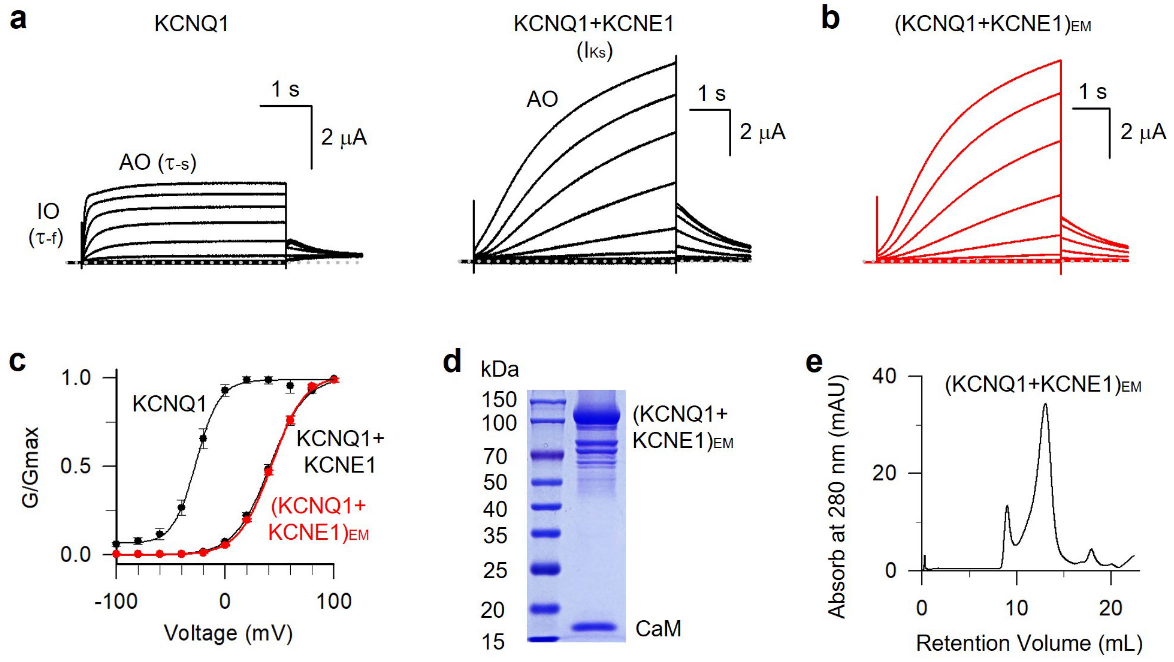

Supplementary information, Fig. S1 KCNE1 significantly modulates the KCNQ1 currents. (a) Representative activation currents of KCNQ1 and KCNQ1+KCNE1 recorded from -120 mV to +80 mV for 4 s or 5 s, and then returned to -40 mV for tail currents. KCNQ1 currents show fast and slow components ($\tau_{\text{-f}}$, $\tau_{\text{-s}}$), approximating currents of the intermediate open (IO) and the activated open (AO) states. **(b)** Representative activation currents of $(\text{KCNQ1+KCNE1})_{EM}$. **(c)** G–V relationships of KCNQ1 (V_{50}= -28.1±0.9 mV, n=6), KCNQ1+KCNE1 (V_{50}= 41.6±2.0 mV, n=8), and $(\text{KCNQ1+KCNE1})_{EM}$ (red, V_{50}=42.1±1.1 mV, n=8). **(d)** SDS-PAGE analysis of the $(\text{KCNQ1+KCNE1})_{EM}$ sample. **(e)** Size-exclusion chromatography of $(\text{KCNQ1+KCNE1})_{EM}$ on Superose 6 (GE Healthcare).
