# Supplementary material for: Secondary structure transitions and dual PIP2 binding define cardiac KCNQ1-KCNE1 channel gating
Source: Cell Res. 2025 Oct 2;35(11):887–99. doi: 10.1038/s41422-025-01182-9 (PMC12589563; doi:10.1038/s41422-025-01182-9)
Supplement: Supplementary file 8 — Supplementary Figure S2 [file 41422_2025_1182_MOESM8_ESM.pdf]

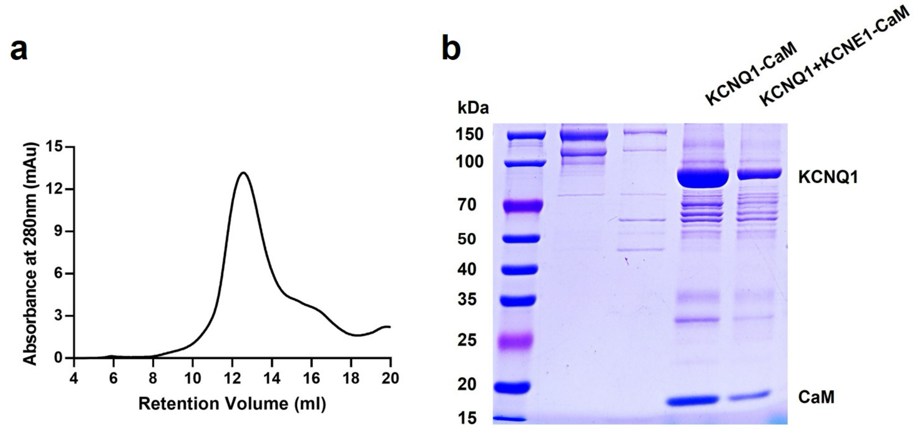

Supplementary information, Fig. S2 Initial attempts with full-length human KCNQ1 co-expressed with KCNE1 yielded only KCNQ1 protein after purification. **(a)** Size-exclusion chromatography of KCNQ1+KCNE1-CaM on Superose 6. **(b)** SDS-PAGE analysis of the KCNQ1+KCNE1-CaM and KCNQ1-CaM.
