# Supplementary material for: Secondary structure transitions and dual PIP2 binding define cardiac KCNQ1-KCNE1 channel gating
Source: Cell Res. 2025 Oct 2;35(11):887–99. doi: 10.1038/s41422-025-01182-9 (PMC12589563; doi:10.1038/s41422-025-01182-9)
Supplement: Supplementary file 9 — Supplementary Figure S3 [file 41422_2025_1182_MOESM9_ESM.pdf]

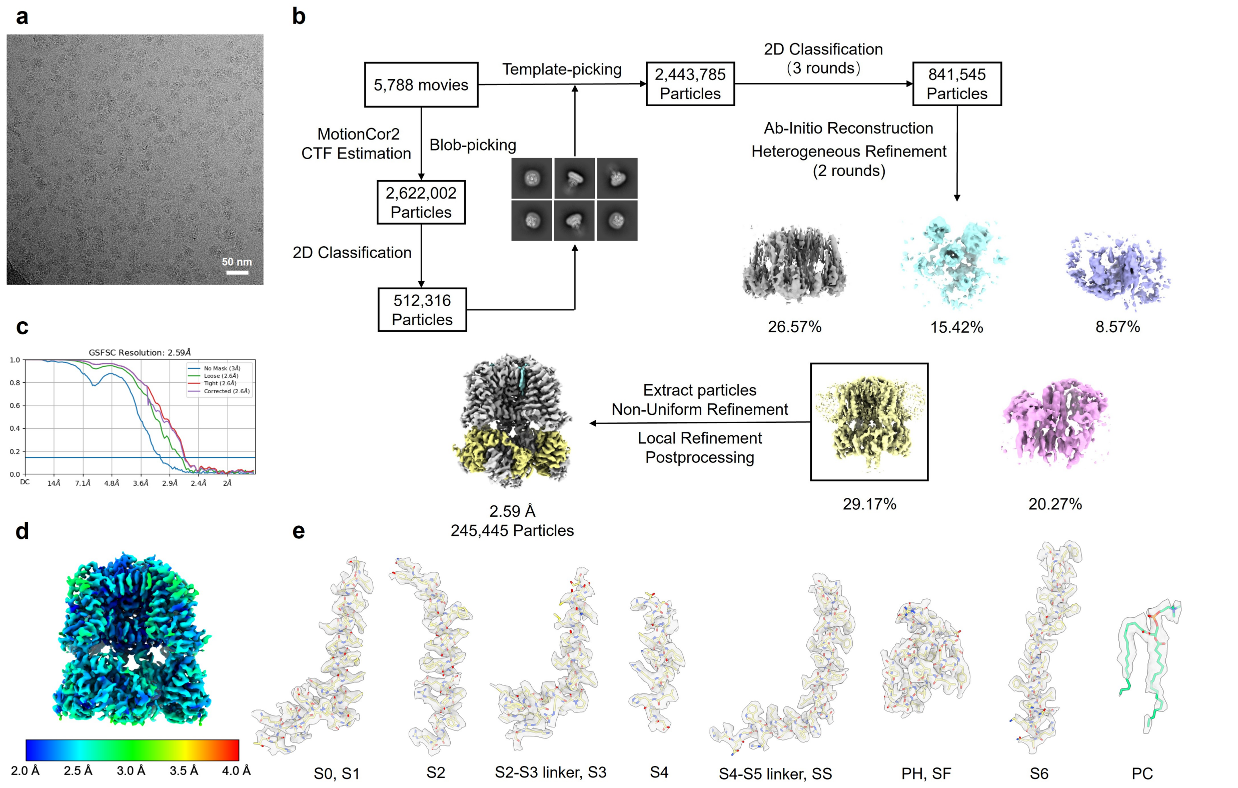

Supplementary information, Fig. S3 Structure determination of hKCNQ1$_{APO}$. (a) A representative cryo-EM micrograph of hKCNQ1$_{APO}$. **(b)** Flowchart of hKCNQ1$_{APO}$ structure determination. **(c)** FSC of the final map. **(d)** Local resolution of the channel complex calculated by Blocres software. **(e)** Cryo-EM densities for various TMs in the hKCNQ1$_{APO}$.
