# Supplementary material for: Secondary structure transitions and dual PIP2 binding define cardiac KCNQ1-KCNE1 channel gating
Source: Cell Res. 2025 Oct 2;35(11):887–99. doi: 10.1038/s41422-025-01182-9 (PMC12589563; doi:10.1038/s41422-025-01182-9)
Supplement: Supplementary file 12 — Supplementary Figure S6 [file 41422_2025_1182_MOESM12_ESM.pdf]

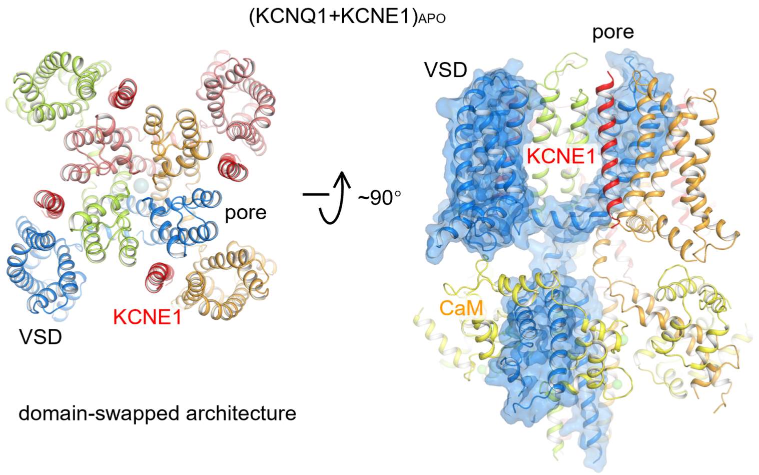

Supplementary information, Fig. S6 Top and side views to show that the KCNQ1+KCNE1 structures consistently follow the domain-swapped architecture. Four subunits are labeled as different colors.
