# Supplementary material for: Secondary structure transitions and dual PIP2 binding define cardiac KCNQ1-KCNE1 channel gating
Source: Cell Res. 2025 Oct 2;35(11):887–99. doi: 10.1038/s41422-025-01182-9 (PMC12589563; doi:10.1038/s41422-025-01182-9)
Supplement: Supplementary file 13 — Supplementary Figure S7 [file 41422_2025_1182_MOESM13_ESM.pdf]

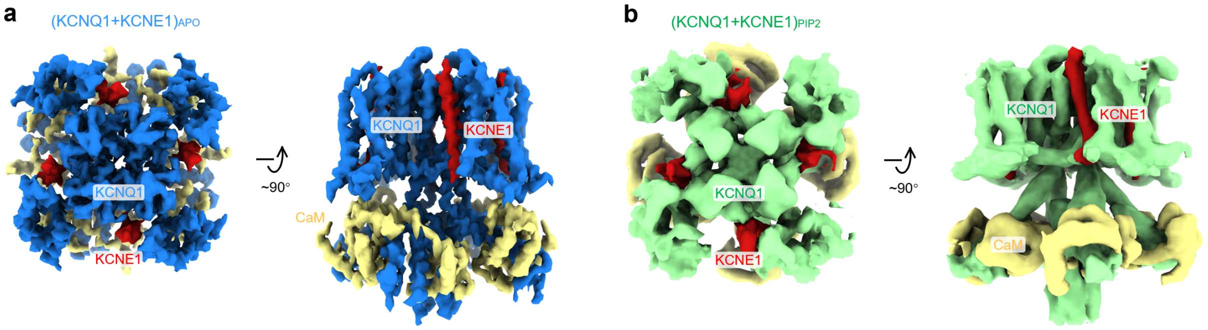

Supplementary information, Fig. S7 Structure determinations of (KCNQ1+KCNE1)$_{APO}$ and (KCNQ1+KCNE1)$_{PIP2}$ in C1 symmetry, confirming the 4:4 stoichiometry between KCNQ1 and KCNE1.
