# Supplementary material for: Secondary structure transitions and dual PIP2 binding define cardiac KCNQ1-KCNE1 channel gating
Source: Cell Res. 2025 Oct 2;35(11):887–99. doi: 10.1038/s41422-025-01182-9 (PMC12589563; doi:10.1038/s41422-025-01182-9)
Supplement: Supplementary file 14 — Supplementary Figure S8 [file 41422_2025_1182_MOESM14_ESM.pdf]

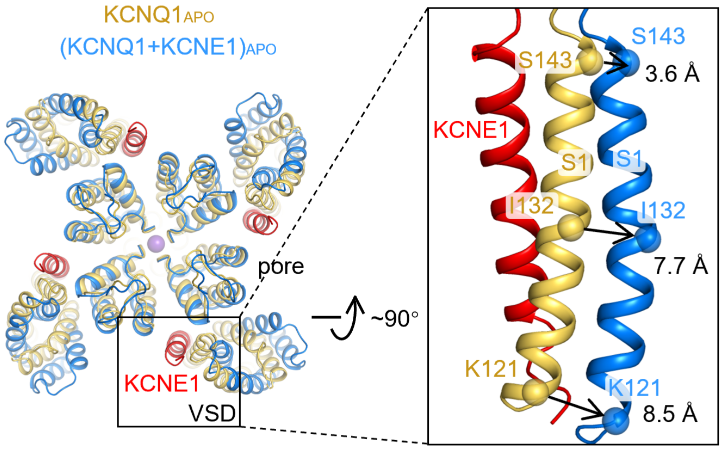

Supplementary information, Fig. S8 KCNE1-induced conformational changes to the VSD. Top and side views of $KCNQ1_{APO}$ and $(KCNQ1+KCNE1)_{APO}$ to show KCNE1 indued a ~10° rotation to the KCNQ1 VSD (counterclockwise from top view), and a significant movement to the S1 segment: K121, I132, and S143 from bottom, middle, and top of S1 show 8.5 Å, 7.7 Å, and 3.6 Å displacement, respectively). Structures were aligned to the filter.
