# Supplementary material for: Secondary structure transitions and dual PIP2 binding define cardiac KCNQ1-KCNE1 channel gating
Source: Cell Res. 2025 Oct 2;35(11):887–99. doi: 10.1038/s41422-025-01182-9 (PMC12589563; doi:10.1038/s41422-025-01182-9)
Supplement: Supplementary file 15 — Supplementary Figure S9 [file 41422_2025_1182_MOESM15_ESM.pdf]

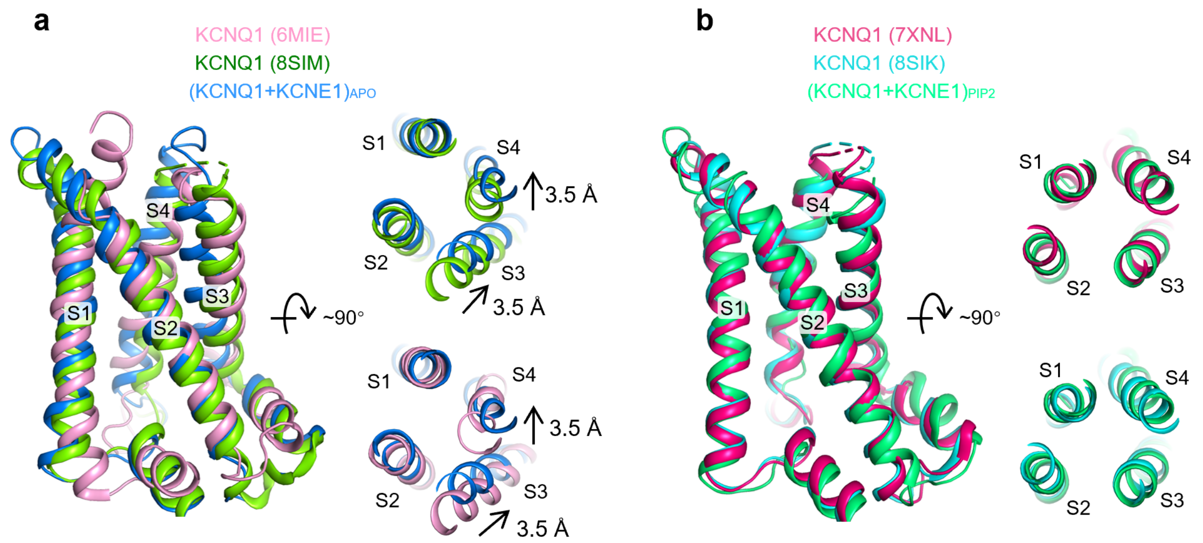

Supplementary information, Fig. S9 KCNQ1 and KCNQ1+KCNE1 show different intermediate and activated VSD structures. (a) Structural comparison of intermediate VSD between KCNQ1 (PDB: 6MIE [1] and PDB: 8SIM[2]) and $(KCNQ1+KCNE1)_{APO}$ exhibited 3.5 Å displacements in S3-S4 while maintaining S1-S2 positions. **(b)** Structural comparison of activated VSD between KCNQ1 (PDB: 7XNL [3] and PDB: 8SIK [2]) and $(KCNQ1+KCNE1)_{PIP2}$ preserved overall S1-S4 positions. To observe potential conformational changes of S3-S4, these VSD structures were aligned to S1-S2.

References

1 Taylor, K. C. *et al.* Structure and physiological function of the human KCNQ1 channel voltage sensor intermediate state. *eLife* **9**, doi:10.7554/eLife.53901 (2020).

2 Mandala, V. S. & MacKinnon, R. The membrane electric field regulates the PIP(2)-binding site to gate the KCNQ1 channel. *Proceedings of the National Academy of Sciences of the United States of America* **120**, e2301985120, doi:10.1073/pnas.2301985120 (2023).

3 Ma, D. *et al.* Structural mechanisms for the activation of human cardiac KCNQ1 channel by electro-mechanical coupling enhancers. *Proceedings of the National Academy of Sciences of the United States of America* **119**, e2207067119, doi:10.1073/pnas.2207067119 (2022).
