# Supplementary material for: Secondary structure transitions and dual PIP2 binding define cardiac KCNQ1-KCNE1 channel gating
Source: Cell Res. 2025 Oct 2;35(11):887–99. doi: 10.1038/s41422-025-01182-9 (PMC12589563; doi:10.1038/s41422-025-01182-9)
Supplement: Supplementary file 16 — Supplementary Figure S10 [file 41422_2025_1182_MOESM16_ESM.pdf]

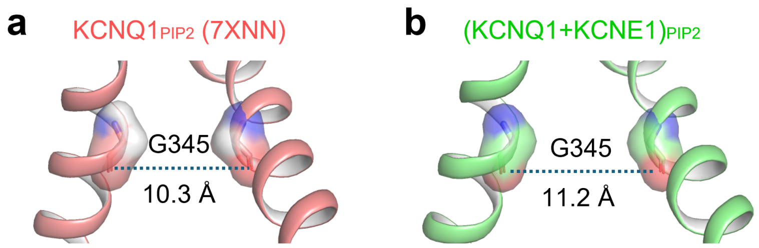

Supplementary information, Fig. S10 Distance analysis of two opposite G345 between KCNQ1 and KCNQ1+KCNE1 in open conformation. G345 in (KCNQ1+KCNE1)$_{PIP2}$ shows larger distance (from 10.3 Å to 11.2 Å) than in KCNQ1$_{PIP2}$ (PDB: 7XNN [1]).

Reference

1 Ma, D. *et al.* Structural mechanisms for the activation of human cardiac KCNQ1 channel by electro-mechanical coupling enhancers. *Proceedings of the National Academy of Sciences of the United States of America* **119**, e2207067119, doi:10.1073/pnas.2207067119 (2022).
