# Supplementary material for: Secondary structure transitions and dual PIP2 binding define cardiac KCNQ1-KCNE1 channel gating
Source: Cell Res. 2025 Oct 2;35(11):887–99. doi: 10.1038/s41422-025-01182-9 (PMC12589563; doi:10.1038/s41422-025-01182-9)
Supplement: Supplementary file 17 — Supplementary Figure S11 [file 41422_2025_1182_MOESM17_ESM.pdf]

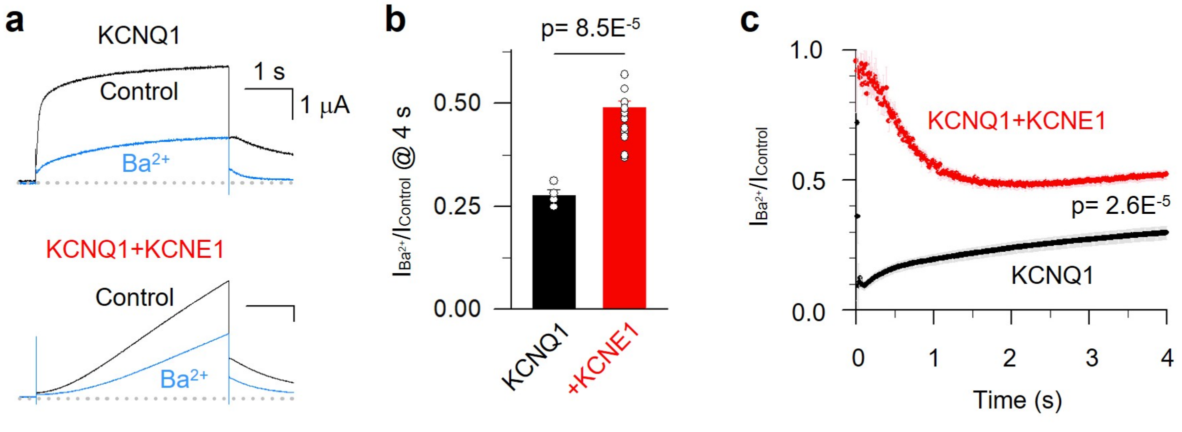

Supplementary information, Fig. S11 Ba^{2+} blockage experiments show KCNQ1 and KCNQ1+KCNE1 channels have different Ba^{2+} sensitivity. (a-b) Representative currents of KCNQ1 and KCNQ1+KCNE1 before and after adding 1 mM Ba^{2+}. The Ba^{2+}-sensitive current $(I_{control}-I_{Ba^{2+}})/I_{control}$ were 0.72±0.01 (n= 4, the $I_{Ba^{2+}}/I_{control}$ ratio was 0.28±0.01) for KCNQ1 and 0.53±0.02 (n=12, the $I_{Ba^{2+}}/I_{control}$ ratio was 0.47±0.02) for KCNQ1+KCNE1 (p= 8.5E-5 with t-test). **(c)** Time-dependence of Ba^{2+} block between KCNQ1 and KCNQ1+KCNE1 channels. The $I_{Ba^{2+}}/I_{control}$ ratio was calculated using testing traces before ($I_{control}$) and after ($I_{Ba^{2+}}$) adding 1 mM Ba^{2+} recorded at 40 mV for 4 seconds (p= 2.6E-5 with t-test).
