# Supplementary material for: Secondary structure transitions and dual PIP2 binding define cardiac KCNQ1-KCNE1 channel gating
Source: Cell Res. 2025 Oct 2;35(11):887–99. doi: 10.1038/s41422-025-01182-9 (PMC12589563; doi:10.1038/s41422-025-01182-9)
Supplement: Supplementary file 18 — Supplementary Figure S12 [file 41422_2025_1182_MOESM18_ESM.pdf]

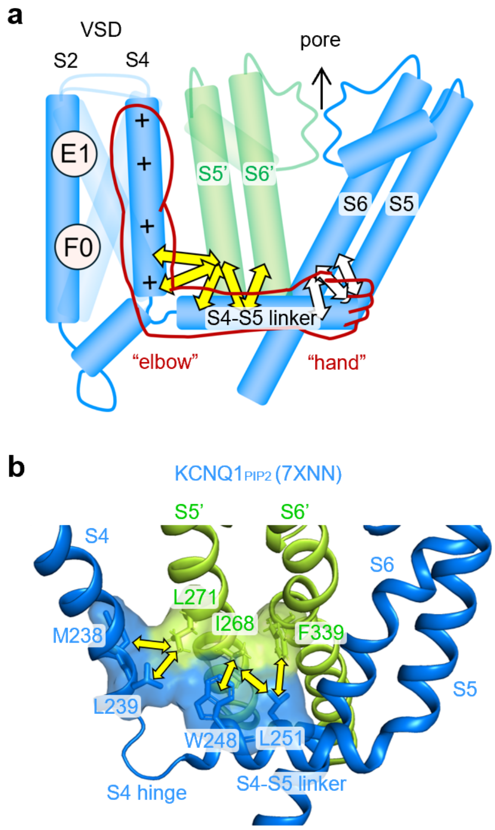

Supplementary information, Fig. S12 The Hand-and-Elbow gating mechanism of KCNQ1. (a) Cartoon scheme illustrating the Hand-and-Elbow gating mechanism of KCNQ1. Only two neighboring subunits (blue and green) from the same tetramer are shown for clarity. **(b)** Five pairs of AO-specific interactions at the elbow site [1] were projected to KCNQ1 (PDB: 7XNN).

Reference

1 Hou, P. *et al.* Two-stage electro-mechanical coupling of a KV channel in voltage-dependent activation. *Nature communications* **11**, 676, doi:10.1038/s41467-020-14406-w (2020).
