# Supplementary material for: Secondary structure transitions and dual PIP2 binding define cardiac KCNQ1-KCNE1 channel gating
Source: Cell Res. 2025 Oct 2;35(11):887–99. doi: 10.1038/s41422-025-01182-9 (PMC12589563; doi:10.1038/s41422-025-01182-9)
Supplement: Supplementary file 19 — Supplementary Figure S13 [file 41422_2025_1182_MOESM19_ESM.pdf]

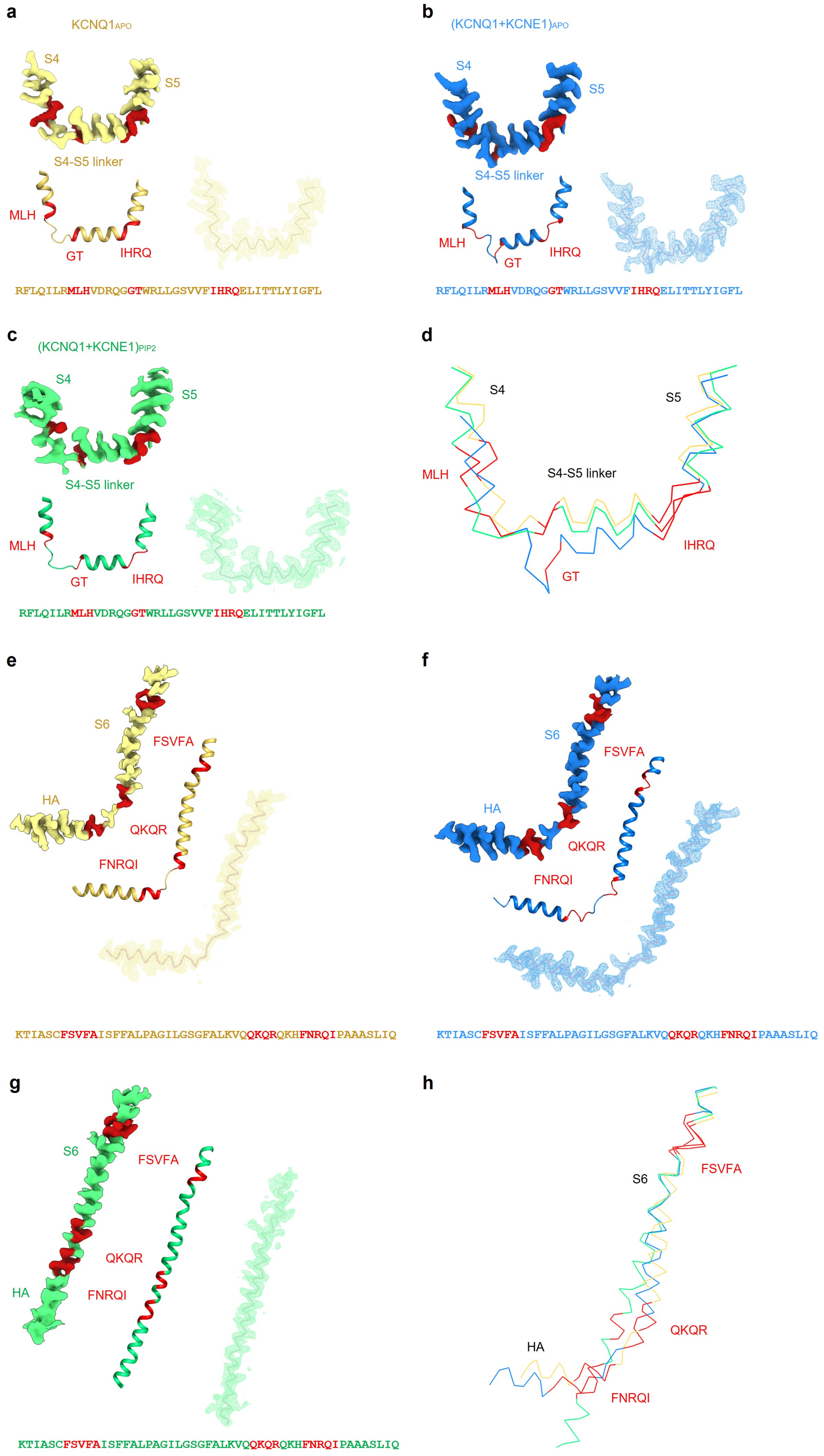
a
KCNQ1APO
S4
S5
S4-S5 linker
MLH
GT
IHRQ
RFLQILRMLHVDRQGGTWRLLGSVVFIHRQELITTLYIGFL
b
(KCNQ1+KCNE1)APO
S4
S5
S4-S5 linker
MLH
GT
IHRQ
RFLQILRMLHVDRQGGTWRLLGSVVFIHRQELITTLYIGFL
c
(KCNQ1+KCNE1)PIP2
S4
S5
S4-S5 linker
MLH
GT
IHRQ
RFLQILRMLHVDRQGGTWRLLGSVVFIHRQELITTLYIGFL
d
S4
S5
MLH
S4-S5 linker
IHRQ
GT
e
S6
FSVFA
HA
QKQR
FNRQI
KTIASCFSVFAISFFALPAGILGSGFALKVQQKQRQKHFNRQIPAAASLIQ
f
S6
FSVFA
HA
QKQR
FNRQI
KTIASCFSVFAISFFALPAGILGSGFALKVQQKQRQKHFNRQIPAAASLIQ
g
S6
FSVFA
QKQR
HA
FNRQI
KTIASCFSVFAISFFALPAGILGSGFALKVQQKQRQKHFNRQIPAAASLIQ
h
FSVFA
S6
QKQR
HA
FNRQI

Supplementary information, Fig. S13 Definition of helix-loop transitions in (KCNQ1+KCNE1)$_{APO}$ and (KCNQ1+KCNE1)$_{PIP2}$. (a-d) Three KCNE1 induced helix-to-loop transitions around the S4-S5 linker: M238/L239/H240 (MLH), G246/T247 (GT), and I257/H258/R259/Q260 (IHRQ). These helix-to-loop transitions preserved during the opening of KCNQ1+KCNE1. **(e-h)** KCNE1 induced three helix-to-loop transitions to S6 and HA: one at the Kink 2, and two at the two ends of the S6/HA linker QKHF (QKQR and FNRQI). These loops undergo loop-to-helix transitions during the opening of S6, following the bend-to-open at Kink1.
