# Supplementary material for: Secondary structure transitions and dual PIP2 binding define cardiac KCNQ1-KCNE1 channel gating
Source: Cell Res. 2025 Oct 2;35(11):887–99. doi: 10.1038/s41422-025-01182-9 (PMC12589563; doi:10.1038/s41422-025-01182-9)
Supplement: Supplementary file 20 — Supplementary Figure S14 [file 41422_2025_1182_MOESM20_ESM.pdf]

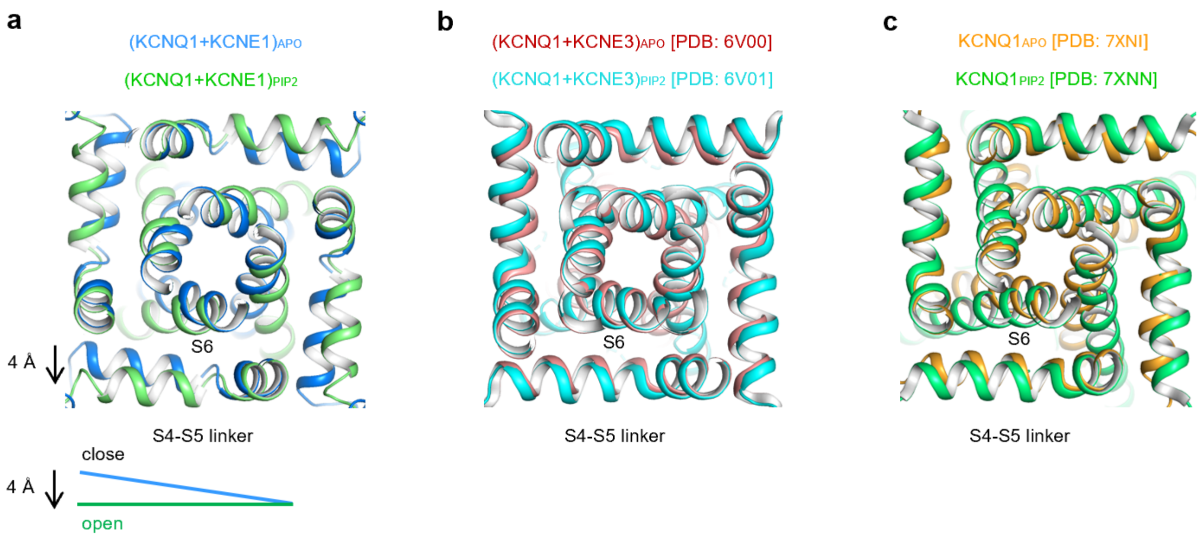

Supplementary information, Fig. S14 KCNQ1+KCNE1 shows different S4-S5 linker motion during channel opening from KCNQ1 and KCNQ1+KCNE3 channels. (a) Structural comparison of the activation gate (S4-S5 linker and S6) between (KCNQ1+KCNE1)$_{APO}$ and (KCNQ1+KCNE1)$_{PIP2}$. The S4-S5 linker shows 4 Å horizontal expansion during the channel opening. **(b)** Structural comparison of the activation gate between (KCNQ1+KCNE3)$_{APO}$ (PDB: 6V00 [1]) and (KCNQ1+KCNE3)$_{PIP2}$ (PDB: 6V01 [1]). The S4-S5 linker shows minimum horizontal expansion during the channel opening. **(c)** Structural comparison of the activation gate between KCNQ1$_{APO}$ (PDB: 7XNI [2]) and KCNQ1$_{PIP2}$ (PDB: 7XNN [2]). The S4-S5 linker shows <1 Å horizontal expansion during the channel opening.

References

1 Sun, J. & MacKinnon, R. Structural Basis of Human KCNQ1 Modulation and Gating. *Cell* **180**, 340-347 e349, doi:10.1016/j.cell.2019.12.003 (2020).

2 Ma, D. *et al.* Structural mechanisms for the activation of human cardiac KCNQ1 channel by electro-mechanical coupling enhancers. *Proceedings of the National Academy of Sciences of the United States of America* **119**, e2207067119, doi:10.1073/pnas.2207067119 (2022).
