# Supplementary material for: Secondary structure transitions and dual PIP2 binding define cardiac KCNQ1-KCNE1 channel gating
Source: Cell Res. 2025 Oct 2;35(11):887–99. doi: 10.1038/s41422-025-01182-9 (PMC12589563; doi:10.1038/s41422-025-01182-9)
Supplement: Supplementary file 21 — Supplementary Figure S15 [file 41422_2025_1182_MOESM21_ESM.pdf]

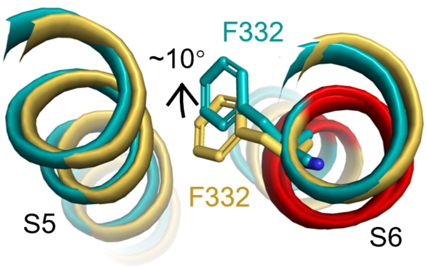

Supplementary information, Fig. S15 F332 motion during KCNQ1 channel gating. From structural comparison between $KCNQ1_{APO}$ and $KCNQ1_{PIP2}$ (PDB: 7XNN [1]), the F332 residue points to the S5, and undergoes a ~10° clockwise rotation during channel opening.

Reference

1 Ma, D. *et al.* Structural mechanisms for the activation of human cardiac KCNQ1 channel by electro-mechanical coupling enhancers. *Proceedings of the National Academy of Sciences of the United States of America* **119**, e2207067119, doi:10.1073/pnas.2207067119 (2022).
