# Supplementary material for: Secondary structure transitions and dual PIP2 binding define cardiac KCNQ1-KCNE1 channel gating
Source: Cell Res. 2025 Oct 2;35(11):887–99. doi: 10.1038/s41422-025-01182-9 (PMC12589563; doi:10.1038/s41422-025-01182-9)
Supplement: Supplementary file 22 — Supplementary Figure S16 [file 41422_2025_1182_MOESM22_ESM.pdf]

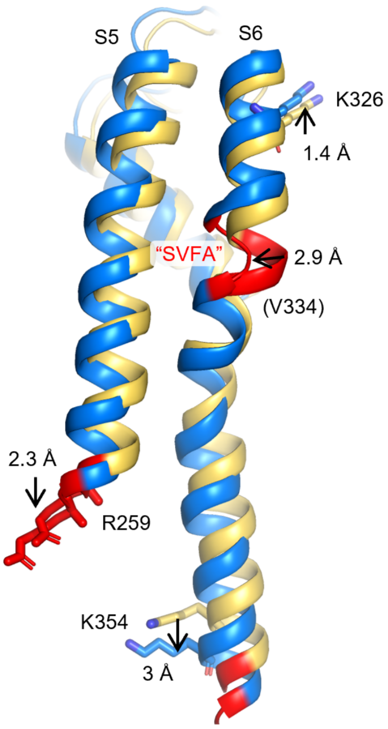

Supplementary information, Fig. S16 KCNE1-induced changes to S5 and S6. KCNE1 induced a 2.9 Å shrink to V334, a 2.3 Å downward movement to the bottom of S5 (R259), and a 3 Å downward movement to the bottom of S6 (K354), and a 1.4 Å upward movement to the top of S6 (K326).
