# Supplementary material for: Secondary structure transitions and dual PIP2 binding define cardiac KCNQ1-KCNE1 channel gating
Source: Cell Res. 2025 Oct 2;35(11):887–99. doi: 10.1038/s41422-025-01182-9 (PMC12589563; doi:10.1038/s41422-025-01182-9)
Supplement: Supplementary file 23 — Supplementary Figure S17 [file 41422_2025_1182_MOESM23_ESM.pdf]

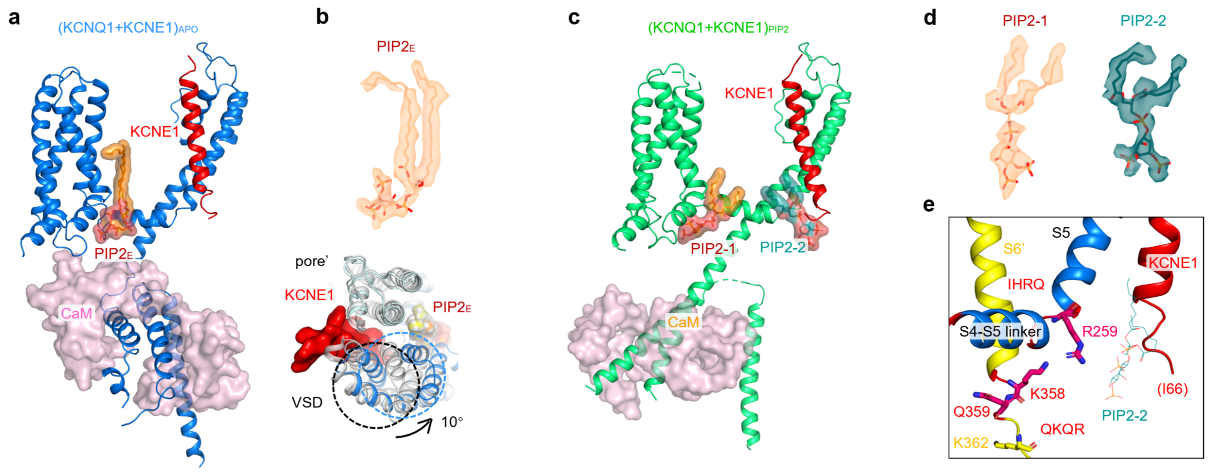

Supplementary information, Fig. S17 Two PIP2 binding sites in KCNQ1+KCNE1. **(a)** In $(KCNQ1+KCNE1)_{APO}$, an endogenous $PIP2_E$ molecule was observed in the canonical PIP2 site. **(b)** The density map of $PIP2_E$ molecule, and the KCNE1-induced ~10° counterclockwise rotation to the VSD may enhance the PIP2-1 sensitivity. **(c)** In $(KCNQ1+KCNE1)_{PIP2}$, two PIP2 molecules (PIP2-1 and PIP2-2) were observed in each KCNQ1 subunit: PIP2-1 was in the canonical site, and PIP2-2 was binding between S4-S5 linker, S6', and KCNE1. **(d)** Density maps of PIP2-1 and PIP2-2 were shown in orange and dark green. **(e)** Residues involved in PIP2-2 binding (R259, K358, Q359, K362, and KCNE1-R67) fall apart in the closed channel structure $(KCNQ1+KCNE1)_{APO}$. PIP2-2 from $(KCNQ1+KCNE1)_{PIP2}$ was also shown as lines.
