# Supplementary material for: Secondary structure transitions and dual PIP2 binding define cardiac KCNQ1-KCNE1 channel gating
Source: Cell Res. 2025 Oct 2;35(11):887–99. doi: 10.1038/s41422-025-01182-9 (PMC12589563; doi:10.1038/s41422-025-01182-9)
Supplement: Supplementary file 24 — Supplementary Figure S18 [file 41422_2025_1182_MOESM24_ESM.pdf]

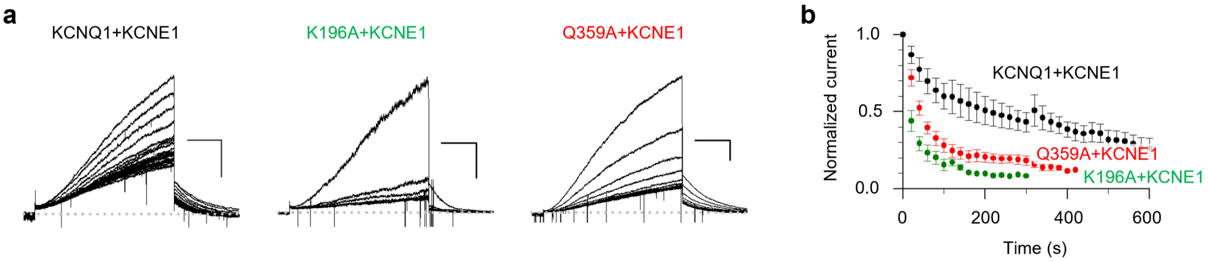

Supplementary information, Fig. S18 K196A+KCNE1 and Q359A+KCNE1 channels show significantly faster current rundown compared to WT I_{Ks}. (a) Current rundown of WT I_{Ks}, K196A+KCNE1, and Q359A+KCNE1 channels. **(b)** Diary plot of current rundown of WT I_{Ks}, K196A+KCNE1, and Q359A+KCNE1 channels. Whole cell patch clamp recordings were performed with the pipette solution removing both PIP2 and ATP molecules to induce current rundown [1,2]. Recordings started immediately following the membrane rupture to form whole cell mode. Currents were tested at 40 mV for 4 s to monitor the current rundown. The current rundown rates are τ_{50}= 245.0±67.4 s for WT I_{Ks} (n=6), τ_{50}= 18.4±2.3 s for K196A+KCNE1 (n=5, p=0.014 with one-way ANOVA), and τ_{50}= 49.3±10.9 s for Q359A+KCNE1 (n=6, p=0.017 with one-way ANOVA).

References

1 Zaydman, M. A. *et al.* Kv7.1 ion channels require a lipid to couple voltage sensing to pore opening. *Proceedings of the National Academy of Sciences of the United States of America* **110**, 13180-13185, doi:1305167110 [pii] 10.1073/pnas.1305167110.

2 Li, Y. *et al.* Intracellular ATP binding is required to activate the slowly activating K+ channel I(Ks). *Proceedings of the National Academy of Sciences of the United States of America* **110**, 18922-18927, doi:1315649110 [pii] 10.1073/pnas.1315649110 (2013).
