# Supplementary material for: Secondary structure transitions and dual PIP2 binding define cardiac KCNQ1-KCNE1 channel gating
Source: Cell Res. 2025 Oct 2;35(11):887–99. doi: 10.1038/s41422-025-01182-9 (PMC12589563; doi:10.1038/s41422-025-01182-9)
Supplement: Supplementary file 25 — Supplementary Table S1 [file 41422_2025_1182_MOESM25_ESM.pdf]

Supplementary Table 1. Cryo-EM data collection, refinement and validation statistics.

Structure	KCNQ1-CaM	KCNQ1-KCNE1-CaM	KCNQ1-KCNE1-CaM-PIP2
EMDB accession code	EMDB-64213	EMDB-63935	EMDB-64038
PDB accession code	9UJ4	9U7F	9UC8
**Data collection and processing**			
Magnification	130,000	130,000	130,000
Voltage (kV)	300	300	300
Electron exposure (e–/Å^2)	49.43	51.45	50.73
Defocus range (μm)	-1.0 ~ -2.0	-1.0 ~ -2.0	-1.0 ~ -2.0
Pixel size (Å)	0.891	0.96	0.96
Symmetry imposed	*C4*	*C4*	*C4*
Initial particle images (#)	2,443,785	1,254,481	5,044,889
Final particle images (#)	245,445	152,699	168,370
Map resolution (Å)	2.59	2.90	3.36
FSC threshold	0.143	0.143	0.143
**Refinement**			
Initial model used (PDB code)	6UZZ	6V00	6V01
Model resolution (Å)	3.10	3.10	3.90
FSC threshold	0.143	0.143	0.143
**Model composition**			
Non-hydrogen atoms	15968	17224	15595
Protein residues	1972	2128	2052
Ligands	16	16	11
***B* factors (Å^2)**			
Protein	54.41	60.35	38.08
Ligand	35.92	101.46	76.76
r.m.s. deviations			
Bond lengths (Å)	0.004	0.007	0.008
Bond angles (°)	0.506	1.109	1.175
**Validation**			
MolProbity score	1.34	1.23	1.99
Clashscore	6.22	2.56	12.19
Poor rotamers (%)	0.48	0.00	0.87
Ramachandran plot			
Favored (%)	99.18	96.85	94.41
Allowed (%)	0.82	3.15	5.39
Disallowed (%)	0.00	0.00	0.20
